# Long noncoding RNA IL6‐AS1 is highly expressed in chronic obstructive pulmonary disease and is associated with interleukin 6 by targeting miR‐149‐5p and early B‐cell factor 1

**DOI:** 10.1002/ctm2.479

**Published:** 2021-07-19

**Authors:** Erkang Yi, Jiahuan Zhang, Mengning Zheng, Yi Zhang, Chunxiao Liang, Binwei Hao, Wei Hong, Biting Lin, Jinding Pu, Zhiwei Lin, Peiyu Huang, Bing Li, Yumin Zhou, Pixin Ran

**Affiliations:** ^1^ National Center for Respiratory Medicine State Key Laboratory of Respiratory Disease & National Clinical Research Center for Respiratory Disease Guangzhou Institute of Respiratory Health The First Affiliated Hospital of Guangzhou Medical University 151 Yanjiang Xi Road Guangzhou Guangdong 510000 China; ^2^ GMU‐GIBH Joint School of Life Sciences Guangzhou Medical University Guangzhou Guangdong 510000 China

**Keywords:** chronic obstructive pulmonary disease, early B‐cell factor 1, hsa‐miR‐149‐5p, IL‐6, IL6‐AS1, inflammation, long noncoding RNA

## Abstract

Chronic obstructive pulmonary disease is a complex condition with multiple etiologies, including inflammation. We identified a novel long noncoding RNA (lncRNA), interleukin 6 antisense RNA 1 (IL6‐AS1), which is upregulated in this disease and is associated with airway inflammation. We found that IL6‐AS1 promotes the expression of inflammatory factors, especially interleukin (IL) 6. Mechanistically, cytoplasmic IL6‐AS1 acts as an endogenous sponge by competitively binding to the microRNA miR‐149‐5p to stabilize IL‐6 mRNA. Nuclear IL6‐AS1 promotes IL‐6 transcription by recruiting early B‐cell factor 1 to the IL‐6 promoter, which increases the methylation of the H3K4 histone and acetylation of the H3K27 histone. We propose a model of lncRNA expression in both the nucleus and cytoplasm that exerts similar effects through differing mechanisms, and IL6‐AS1 probably increases inflammation via multiple pathways.

## Graphical abstract

1. The study provides the evidence that a novel lncRNA IL6‐AS1 is highly expressed in COPD and is associated with inflammation.
2. IL6‐AS1 acts as the competitive endogenous RNA (ceRNA) to competitively bind to hsa‐miR‐149‐5p to regulate IL‐6 expression.
3. IL6‐AS1 acts as a guide to promote the gene expression via recruiting EBF1 to the region of the IL‐6 promoter.

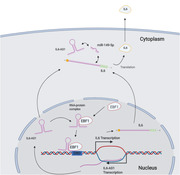

**Abbreviations**
ceRNA: competing endogenous RNA
ChIP: chromatin immunoprecipitation
COPD: chronic obstructive pulmonary disease
EBF1: early B‐cell factor 1
ENCODE: Encyclopedia of DNA Elements
FISH: fluorescence in situ hybridization
GO: gene ontology
GSEA: gene‐set enrichment analysis
IL‐6: interleukin 6
IL6‐AS1: interleukin 6 antisense RNA 1
ISH: in situ hybridization
KEGG: Kyoto Encyclopedia of Genes and Genomes
RACE: 5′/3′ rapid amplification of cDNA ends
RIP: RNA immunoprecipitation
siRNA: small interfering RNAs
SS: smart silencer

## 1 INTRODUCTION

It has been proposed worldwide that chronic obstructive pulmonary disease (COPD) is characterized by progressive airflow obstruction and continuously aggravated airflow limitation with an abnormal and persistent inflammatory response of the lungs or airways to cigarette smoking or harmful gases and particles.^1^ COPD affects approximately 13.7% individuals aged 40 years or older,^2^ and caused more than 0.9 million deaths in China, which was the third leading cause of death in 2013.^3^ According to World Health Organization (WHO), COPD affects about 251 million population and causes over 3 million deaths in the world every year.^4^

The prominent pathological features of COPD are respiratory bronchiolitis, parenchymal destruction (emphysema), and mucus hypersecretion, and the primary etiopathogenetic mechanisms involved appear to be chronic inflammation, accelerated aging, and oxidative stress.^5^ Chronic airway inflammation is characterized by increased proinflammatory mediators such as cytokines, chemokines, growth factors, and lipid mediators secreted by alveolar macrophages, neutrophils, T lymphocytes, innate lymphoid cells, and structural cells.^6^

Interleukin (IL) 6 is a key proinflammatory cytokine and modulates the development of various human diseases, including COPD. Two previous studies reported elevated serum levels of IL‐6 were predictive of increased mortality in COPD patients.^6^, ^7^ However, the mechanisms that drive excessive IL‐6 production in COPD are poorly understood, and the molecular pathways involved are rarely reported.^8^, ^9^

Only 1.2% of human genes encode proteins, while much of the genetic sequence contains long noncoding RNAs (lncRNAs).^10^, ^11^ Growing evidence has reported that lncRNAs are transcripts longer than 200 nucleotides^12^ with regulatory function in human health and disease.^13^, ^14^ lncRNAs act as scaffolds for chromatin modifiers and as transcriptional regulators of proteins and microRNAs (miRNAs).^15^, ^16^ lncRNA function is typically mediated through complex formation with proteins, RNA, or DNA^17^, ^18^ and affects biological and pathological processes.

Through a high‐throughput sequencing of samples, the expression of hundreds of lncRNAs vary considerably between COPD patients and smokers with non‐COPD and notably that the lncRNA COPDA1 is significant highly expressed in COPD and facilitates proliferation of human bronchial smooth muscle cells by regulating membrane spanning four domains A1.^19^ However, only a few reports have described inflammation from the perspective of lncRNA regulation.^20^ In the present work, we report that an antisense lncRNA termed interleukin 6 antisense RNA 1 (IL6‐AS1) is highly expressed in COPD and correlates with IL‐6 expression, acting as a miRNA sponge by competitively binding to miR‐149‐5p to upregulate IL‐6 in the cytoplasm. Furthermore, we also found that IL6‐AS1 regulates IL‐6 promoter activity and histone modification by recruiting the early B‐cell factor 1 (EBF1) transcription factor, which increases IL‐6 expression and subsequently airway inflammation. This research elucidates a mechanism of lncRNA‐mediated inflammation and the key player of IL6‐AS1 in COPD.

## 2 MATERIALS AND METHODS

### 2.1 Clinical specimens

Thirty‐five patients were recruited as previously described at the First Affiliated Hospital of Guangzhou Medical University.^19^ Lung specimens were obtained from these 35 patients, 19 of whom had COPD and 16 of whom did not have COPD. The patients underwent lung cancer resection or lung volume reduction surgery. The study was approved by the Research *Ethics Committee* of the First Affiliated Hospital of Guangzhou Medical University (No. 2013‐38).

All the patients gave written informed consent for providing lung tissue samples.

### 2.2 Cell culture

Primary human bronchial fibroblasts (HBFs) and fibroblast medium were purchased from ScienCell (USA). HBFs were cultured in fibroblast medium containing 2% fetal bovine serum (FBS, ScienCell), 1% penicillin‐streptomycin (ScienCell), and 1% fibroblast growth supplement (ScienCell). Cells at passages 4–8 were used for the experiments. Human lung fibroblast 1 (HFL1, ATCC CCL‐153) cells and F‐12K (Kaighn's modification of Ham's F‐12 medium, ATCC 30‐2004) were purchased from ATCC (Manassas, VA, USA). HFL1 were cultured in F‐12K containing 10% FBS (Gibco, Thermo Fisher Scientific, Waltham, MA, USA) and 1% penicillin‐streptomycin (Invitrogen, Carlsbad, CA, USA). Primary human bronchial smooth muscle cells (HBSMCs) and smooth muscle cell medium were purchased from ScienCell (Carlsbad, CA, USA). HBSMCs were cultured in smooth muscle cell medium containing 2% FBS (ScienCell), 1% penicillin‐streptomycin (ScienCell), and 1% smooth muscle cell growth supplement (ScienCell). BEAS‐2B (ATCC CRL‐9609) cells and RPMI‐1640 Medium (ATCC 30‐2001) were purchased from ATCC. HFL1 were cultured in RPMI‐1640 Medium containing 10% FBS (Gibco, USA) and 1% penicillin‐streptomycin (Invitrogen, USA). Above primary cells or cell lines were all cultured and incubated at 37°C under a humidified atmosphere containing 5% CO2.

### 2.3 Transfection

HBF and HFL1 cells were transfected with small interfering RNAs (siRNAs) or plasmids using Lipofectamine RNAi Max (Invitrogen) according to the manufacturer's instructions. siRNAs of IL6‐AS1 and a lentiviral vector overexpressing IL‐6 were purchased from GenePharma (Shanghai, China). siRNAs of EBF1 and IL6‐AS1 Smart Silencer were designed and constructed from RiboBio (Guangzhou, China). Overexpression vectors for IL6‐AS1 and EBF1 were purchased from Genechem (Shanghai, China). For each siRNA or Smart Silencer transfection, 100,000 HBF or HFL1 cells were seeded per well in 12‐well plates 12–16 h before transfection. siRNAs and Smart Silencers against IL6‐AS1 or EBF1 were used for transfection at a final concentration of 5 nM. Forty‐eight hours after transfection, cells were harvested for experiments. The IL6‐AS1 overexpression lentivirus in the LV5 vector and the IL6‐AS1‐RNA interference lentivirus in the LV3 vector were purchased from GenePharma. For lentivirus‐mediated overexpression or knockdown, HBF and HFL1 cells were transduced with LVIL6‐AS1 or KDIL6‐AS1 at a final concentration of 5 MOI at 50%–60% confluence. Seventy‐two hours after infection, cells were harvested for experiments.

### 2.4 RNA extraction, cDNA synthesis, and qRT‐PCR

Total cellular and tissue RNA was extracted using Trizol reagent (Invitrogen). cDNA from 1000 ng total RNA was synthesized with a PrimeScript RT reagent kit with gDNA Eraser (Takara, Japan). qRT‐PCR was conducted using TB Green Premix Ex Taq II (Tli RNaseH Plus, Takara) and analyzed on a CFX Connect real‐time PCR detection system (Bio‐Rad, USA). The relative expression levels of the unigenes were calculated using the 2^−∆∆^CT method, with GAPDH as an internal control. All specific primers used in the study are listed in Table S1.

### 2.5 In situ hybridization and RNA fluorescence in situ hybridization (FISH)

A locked nucleic acid (LNA) probe with complementarity to a section of IL6‐AS1 was labeled with 5′‐digoxigenin and 3′‐digoxigenin and synthesized by Biosense Bioscience Co. (Guangzhou, China). Following deparaffinization and rehydration, the tissue sections were incubated with proteinase K for 20 min at 37°C. Then the samples were fixed in 4% paraformaldehyde, hybridized with the DIG‐IL6‐AS1 probe at 55°C overnight. The next day, samples were incubated overnight at 4°C with anti‐digoxigenin monoclonal antibody (Roche, Switzerland), and were incubated by using streptavidin–biotin–peroxidase complex for 20 min. Finally, sections were washed with PBS three times (5 min/each), mounted on coverslips, and observed.

A FISH kit (RiboBio) was used according to the manufacturer's protocol. HBF and HFL1 cells were seeded in 24‐well plates, and cells were washed with PBS and fixed with 4% formaldehyde in PBS (pH 7.4) for 20 min at room temperature. Next, cells were washed in PBS three times (5 min/each) and permeabilized with PBS containing 0.1% Triton X‐100 at 4°C for 10–15 min, then washed again with PBS three times (5 min/each). The cells were hybridized with 5 μM CY3 probe overnight. The CY3‐labeled FISH probe for IL6‐AS1 was provided by RiboBio. Next, the cells were washed for 5 min in 4× standard saline citrate (SSC) at 42°C three times and then washed in 2× SSC and 1× SSC once at 42°C. Finally, cells were washed with PBS one time for 5 min. Cells were mounted on coverslips, counterstained with DAPI, and observed.

### 2.6 Nuclear–cytosol fractionation

We used a PARIS kit according to the manufacturer's protocol. HBF and HFL1 cells (1 × 10^7^) were collected and wash by PBS, the cell pellet was resuspended with 400 μl of ice‐cold cell fractionation buffer on ice for 10–15 min. Samples were centrifuged for 5 min at 4°C and 500 × *g*, and carefully aspirated the cytoplasmic fraction away from the nuclear pellet in a clear tube. Then, 300 μl cell disruption buffer nuclear pellet was added. Next, nuclear samples were vortexed every 10 min for 10 s to ensure complete lysis. After that an equal volume of lysis/binding solution was added into the cytoplasmic fraction and the nuclear lysate, which will be used for RNA isolation. We then used reverse transcription and qRT‐PCR for RNA.

### 2.7 5′/3′ Rapid amplification of cDNA ends (RACE)

RACE assays were performed to determine the transcriptional initiation and termination sites of IL6‐AS1 using a SMARTer RACE 5′/3′ kit (Takara) according to the manufacturer's instructions. The sequences for the IL6‐AS1‐specific nested PCR primers used are provided in Table S2. Briefly, total RNA was extracted from HBF cells using Trizol reagent (Invitrogen) and treated with DNase I, followed by SMARTer first‐strand cDNA synthesis to generate 5′‐RACE‐ or 3′‐RACE‐ready cDNA that was then amplified using multiple primers. PCR fragments were purified and cloned into the pMD19‐T vector using the In‐Fusion HD cloning reagent provided in the kit. The plasmids containing the inserts were subsequently sequenced using the *standard Sanger* method.

### 2.8 ELISA analysis and Bio‐Plex assay

The cell culture supernatant was collected by centrifugation at 2000 ×  *g* for 10–15 min at room temperature, and secreted IL‐6 was quantified using the Human IL‐6 DuoSet ELISA (R&D Systems, USA) according to the manufacturer's instructions. Cytokine levels were measured on the Bio‐Plex 200 suspension array system (Bio‐Rad) with the Bio‐Plex pro human cytokine 9‐plex assay (Bio‐Rad).

### 2.9 Western blotting

Cells were lysed for 30 min at 4°C in RIPA lysis buffer (89901, Thermo Fisher Scientific) containing a protease inhibitor cocktail (78430, Thermo Fisher Scientific). Protein extracts were resolved through 10% SDS‐polyacrylamide gel electrophoresis, transferred to polyvinylidene difluoride membranes (Bio‐Rad), and probed with antibodies against human EBF1 (SAB2501166, Sigma‐Aldrich, St. Louis, MO, USA), p50/p65 (ab32360, Abcam, Cambridge, UK), p65 (ab32536, Abcam), GAPDH (10494‐1‐AP, Proteintech, Rosemont, IL, USA), and laminB1 (ab8982, Abcam) and then with a peroxidase‐conjugated secondary antibody (Proteintech). Cells were then visualized using chemiluminescence by Amersham Imager 680 (Thermo Fisher Scientific).

### 2.10 Poly‐A mRNA sequencing and bioinformatics

Sequencing of knockdown IL6‐AS1 was performed at BGI (Shenzhen, China) using the BGISEQ‐500 system. The RNA‐seq data were aligned to the Ensembl v84 transcript annotations. DEmRNAs were subjected to gene ontology (GO) functional enrichment (http://geneontology.org/) and Kyoto Encyclopedia of Genes and Genomes (KEGG) pathway analysis (http://www.kegg.jp/). The expression matrix of filtered genes was subjected to gene‐set enrichment analysis (GSEA, http://software.broadinstitute.org/gsea/index.jsp). Both Coding Potential Calculator 2 (http://cpc2.cbi.pku.edu.cn/) and txCdsPredict from UCSC and phyloCSF were used to calculate the coding potential of IL6‐AS1. Two sequencing databases of human COPD samples, GSE38974^21^ and GSE76925^22^ were downloaded from the Gene Expression Omnibus (GEO) database. Limma in R was used to identify differentially expressed genes. The secondary structure of IL6‐AS1 was predicted using RNAfold (http://rna.tbi.univie.ac.at/cgi‐bin/RNAWebSuite/RNAfold.cgi) via centroid plain structure and minimum free energy plain structure. Associated transcription factors of the IL‐6 promoter were predicted by JASPAR (http://jaspar.genereg.net/) and visualized in the UCSC genome browser (https://genome.ucsc.edu/). PPI network of 62 genes was constructed by using STRING database (http://string‐db.org) and was visualized with Cytoscape (v.3.6.1). The top 10 genes were obtained by cytohubba. Associated histone modification was defined by chromatin immunoprecipitation (ChIP)‐seq from IMR‐90 in the Encyclopedia of DNA Elements (ENCODE, https://www.encodeproject.org/) and visualized in the UCSC genome browser.

### 2.11 Luciferase assay

The wild‐type and mutant of human IL‐6 3′UTR and IL6‐AS1 were amplified from human genomic DNA and individually inserted into GV306 (Genechem) using the XhoI and NotI sites. Similarly, the fragment of IL‐6 3′UTR or IL6‐AS1 mutant was inserted into the GV306 control vector at the same sites. The IL‐6 wild‐type was obtained by cloning a 2100‐bp DNA fragment (−2000 to +100 with respect to the IL‐6 transcriptional starting site) into GV238 upstream of the luciferase reporter gene. The mutants IL‐6‐mut1 and IL‐6‐mut2 were generated by mutating the EBF1 recognizing motif. The pRL‐TK plasmid delivering *Renilla* luciferase was cotransfected as a control. Luciferase activity was measured 48 h posttransfection using a dual‐glo luciferase reporter system according to the manufacturer's instructions (Promega, Madison, WI, USA). Firefly luciferase units were normalized against *Renilla* luciferase units to control for transfection efficiency.

### 2.12 RNA immunoprecipitation

We performed RNA immunoprecipitation (RIP) using a Magna RIP RNA‐binding protein immunoprecipitation kit (Millipore Sigma 17–700, Burlington, MA, USA) according to the manufacturer's instructions. Briefly, 1 × 10^7^ cells for each IP sample were harvested and lysed with RIP lysis buffer for 10 min at 4°C. The cell extract was centrifuged for 10 min at 14,000 × *g* and 4°C. The supernatant was divided into two and anti‐EBF1 antibody (SAB2501166, Sigma‐Aldrich) or anti‐Ago2 antibody (ab186733, Abcam) and IgG were added. The 50 μl of protein A/G agarose beads were then added to each tube followed by rotation at 4°C overnight. A magnetic frame was used to remove the supernatant, followed by six washes with a lysis buffer. Protease K and RNase inhibitor were added to the lysis buffer, followed by rotation for 30 min at 55°C to remove the protein and eluted RNA‐protein complex. Phenol‐chloroform (125:24:1) was added to the remaining solution at −80°C overnight, and RNA was then extracted. qRT‐PCR was used to assess the expression of IL6‐AS1, IL‐6, and miR‐149‐5p in the immunocomplex.

### 2.13 RNA pull‐down assay and mass spectrum

Full‐length IL6‐AS1 and antisense IL6‐AS1 sequences were prepared by in vitro transcription using a TranscriptAid T7 high yield transcription kit (Thermo Fisher Scientific K0441), treated with RNase‐free DNase I, and purified with a GeneJET RNA purification kit (Thermo Fisher Scientific K0731). Extracts were prepared with RIPA lysis buffer (89901, Thermo Fisher Scientific). RNA pull‐down was performed with a Magnetic RNA‐Protein Pull‐Down Kit (Pierce 20164, Rockford, IL, USA) according to manufacturer's instructions. Three micrograms of biotin‐labeled RNA and 1 mg of nuclear extract were used in each pull‐down assay. The protein retrieved was measured by standard immunoblotting. Silver staining was performed with a Fast Silver Stain Kit (P0017S, Beyotime, Beijing, China) following the manufacturer's recommendations. The bands were subjected to protein identification by mass spectrometry sequence prediction.

### 2.14 ChIP

ChIP was performed using a SimpleChIP Enzymatic Chromatin IP Kit (Cell Signaling Technology, Danvers, MA, USA) according to the manufacturer's instructions. HBF cells (4 × 10^6^) for each ChIP sample were washed with PBS and incubated for 10 min with the final concentration of 1% formaldehyde at room temperature. Cross‐linking was halted with 0.1 M glycine for 5 min. The cells were washed twice with ice‐cold PBS and lysed with a lysis buffer containing PIC for 10 min at 4°C, then cross‐linked chromatin was sonicated into fragments with an average length of 200–1000 bp. Next, 5 μg of specific antibodies were added into samples and rocked for overnight at 4°C. Immunoprecipitated DNA was purified using DNA purification columns and collection tubes (Cell Signaling Technology). The final ChIP DNA was then used as a template in qPCR with the primers listed in Table S1. ChIP‐grade anti‐Rpb1 CTD antibody (Cell Signaling Technology 2629S), anti‐EBF1 antibody (SAB2501166, Sigma‐Aldrich), anti‐methyl‐histone H3 (Lys4) antibody (Cell Signaling Technology 5326S), anti‐tri‐methyl‐histone H3 (Lys4) antibody (Cell Signaling Technology 9751S), acetyl‐histone H3 (Lys27) (Cell Signaling Technology 8173S), and anti‐IgG (Cell Signaling Technology 2729) were used in this study.

### 2.15 Immunofluorescence

Cells were fixed with 4% paraformaldehyde in PBS for 20 min and treated with 0.1% Triton X‐100 in PBS for 5 min. The cells were then incubated with rabbit anti‐human monoclonal antibody against EBF1 (1:200, ab108369, Abcam) overnight at 4°C, followed by incubation with Alexa Fluor 488 goat anti‐rabbit IgG (H+L) (1:500, Molecular Probes, Invitrogen) for 60 min. Images were captured using an Axio Observer 7 (Zeiss, Oberkochen, Germany).

### 2.16 Chromatin isolation by RNA purification (ChIRP)

ChIRP assays were performed as previously described.^23^ ChIRP probes against IL6‐AS1 and the LacZ probe were designed and synthesized by GZSCBIO (China). HBF cells were collected by lysis buffer. The lysates were sonicated by Cole‐Parmer 130 (USA) on ice and were sonicated for 10‐s intervals and 15‐s breaks for a total of 20 min. Then, the sonicated cell lysate was mixed with the biotinylated DNA probe mixture for lncRNA IL6‐AS1 and hybridized overnight at 4°C. The binding complexes were covered with streptavidin‐conjugated magnet beads. RNA was eluted with Eluent Buffer (0.2U/μl Proteinase K), and qRT‐PCR assay was performed to assess the enrichment of miR‐149‐5p associated with IL6‐AS1.

### 2.17 Statistical analysis

The significance between means was determined by two‐tailed paired Student's *t*‐test, one‐way analysis of variance (ANOVA) test, or with two‐way analysis of variance (ANOVA) test. All *p*‐values are represented as follows: ns (not significant), *p < 0.05, **p < 0.01, and ***p < 0.001. All statistical analyses were performed with GraphPad Prism v8.0.1.

## 3 RESULTS

### 3.1 lncRNA IL6‐AS1 is upregulated in COPD and associated with inflammation

**FIGURE 1:**
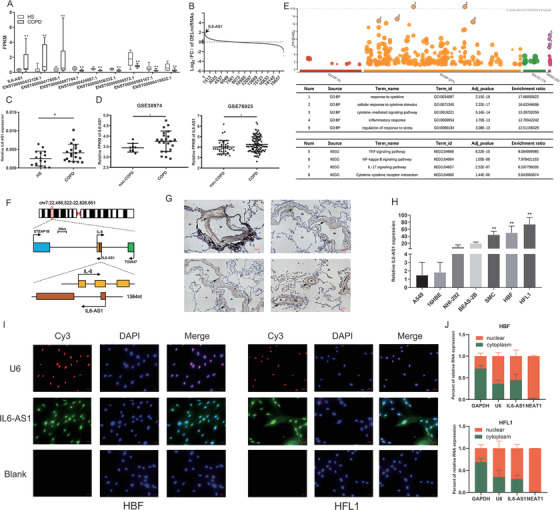
Long noncoding RNA (lncRNA) IL6‐AS1 is overexpressed in chronic obstructive pulmonary disease (COPD) and mainly localized in HBF and HFL1 cells. (A) RNA‐seq analysis of lung tissues from COPD patients and smokers with non‐COPD identified nine lncRNAs overexpressed in COPD (ENST00000417650, ENST00000424887, ENST00000433126, ENST00000556332, ENST00000585167, ENST00000606572, ENST00000607744, and ENST00000424887.1). (B) lncRNA IL6‐AS1 is more differentially expressed (higher fold‐change) as compared to other lncRNAs. (C) qRT‐PCR analysis of the lncRNA IL6‐AS1 in samples from 17 smokers with non‐COPD and 19 COPD patients (two‐tailed *t*‐test). HS: healthy smokers. (D) IL6‐AS1 expression in samples from the Institute for Systems Biology (Seattle, WA, USA) and Brigham and Women's Hospital (Boston, MA, USA) (limma package). (E) Gene ontology (GO) and KEGG pathway enrichment analysis were performed with the lncRNA‐mRNA coexpression network. (F) Schematic illustration indicating genomic location of IL6‐AS1 and IL6. (G) Representative images of COPD tissues after H&E and in situ hybridization (ISH staining for IL6‐AS1. The brown color represents the positive ISH staining, the blue represents nuclear staining. Al: alveoli; Br: bronchus. (H) qRT‐PCR analysis of IL6‐AS1 expression in various airway structure cells, including A549, 16HBE, NHI‐292, BEAS‐2B, smooth‐muscle cells, HBF, and HFL1 (one‐way ANOVA, *n* = 4 biological replicates). (I) Fluorescence in situ hybridization analysis of the subcellular distribution of IL6‐AS1 in HBF and HFL1 cells. Green, IL6‐AS1; Red, U6; Blue, nuclear staining. (J) Nuclear fractionation analysis and qRT‐PCR analysis of IL6‐AS1 expression in the nucleus and cytoplasm. Error bars represent means ± SD, using three biological replicates (*n* = 4 biological replicates). Error bars represent means ± SD. *p < 0.05 and **p < 0.01

To identify critical lncRNAs that were potentially involved in the COPD progression, we collected clinical lung tissue samples from non‐COPD and COPD patients for RNA‐seq in our previous work.^19^ Nine lncRNAs were consistently differentially expressed in samples from smokers with COPD as indicated (Figure 1A), and only IL6‐AS1 with high even in fold‐change of expression among differentially expressed lncRNAs (Figure 1B) was significantly upregulated in the subsequent verification between samples from 17 smokers without COPD and 19 smokers with COPD (Figure 1C, Figure S1 and Table S1). Moreover, similar RNA‐seq matrices of COPD lung tissue from the Gene Expression Omnibus (including GSE38974^21^ and GSE76925^22^) showed significant overexpression of IL6‐AS1 in COPD patients (Figure 1D).

Next, we used our RNA‐seq matrix to establish lncRNA‐mRNA coexpression network by WGCNA,^24^ and searched for mRNAs correlated with IL6‐AS1 (correlation coefficient >0.8; Figure S2A). In total, 791 potential target mRNAs were found, and GO and KEGG^25^ analysis showed high enrichment in response to cytokines and inflammation as indicated, suggesting that IL6‐AS1 function is highly correlated with inflammation (Figure 1F). GSEA^26^ yielded similar conclusions (Figure S2B). We then confirmed that IL6‐AS1 was a noncoding RNA by using two databases^27^ (Figure S2C,D). Genotype‐tissue expression analysis^28^ found that IL6‐AS1 expression in lung tissue was second only to expression in adipose and spleen tissue (Figure S2E).

IL6‐AS1 is located on human chromosome 7p15.3 and has two exons (Figure 1F). Then for defining the cellular location of IL6‐AS1 in lung tissue, ISH experiment was performed by using COPD lung sections and found IL6‐AS1 was mainly expressed in lung fibroblasts (Figure 1G). To validate this finding, we measured the expression of IL6‐AS1 in the lung cell lines A549, 16HBE, NHI‐292, BEAS‐2B, HBSMC, HBF, and HFL1 (Figure 1H); the results showed that IL6‐AS1 was highly expressed in lung fibroblasts. We therefore selected HBF and HFL1 cells for functional research. Next, the full length of IL6‐AS1 was verified and there were 62 more bases at the 5′‐end and 18 more bases at the 3′‐end of IL6‐AS1, with a total length of 1444 nt, the remaining bases were consistent with structures in the NCBI database (Figure S3). Recent studies have shown that the functional mechanism of lncRNAs is closely related to subcellular localization.^29^, ^30^ The results of FISH experiment indicated that IL6‐AS1 was expressed both in the nucleus and cytoplasm (Figure 1I). For validation, we extracted RNA from the nucleus and cytoplasm and used qRT‐PCR to measure IL6‐AS1 expression, with similar findings (Figure 1J). These suggested that IL6‐AS1 may contribute to the development and/or progression of COPD and should therefore be characterized in detail.

### 3.2 IL6‐AS1 promotes expression of inflammatory factors such as IL‐6

**FIGURE 2:**
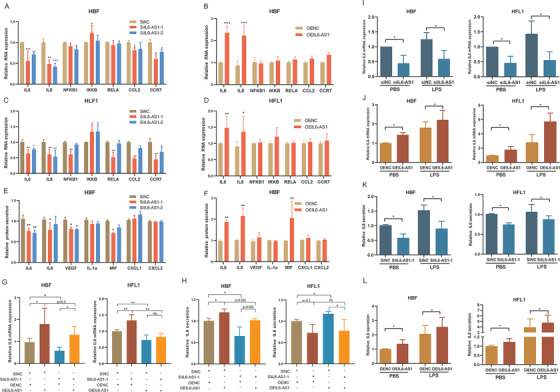
IL6‐AS1 promotes the expression of inflammatory cytokines. (A and B) qRT‐PCR analysis of relative gene expression (including IL‐6, IL‐8, NFκB1, IKκB, RELa, CCL2, and CCR7) in HBF cells (two‐way ANOVA, *n* = 3 biological replicates). (C and D) qRT‐PCR analysis of relative gene expression as indicated in HFL1 cells (two‐way ANOVA, *n* = 3 biological replicates). (E and F) Multiplex immunoassay analysis of protein expression of nine cytokines and chemokines (including IL‐6, IL‐8, VEGF, IL‐1α, MIF, CXCL1, and CXCL2) regulated by IL6‐AS1 (two‐way ANOVA, *n* = 3 biological replicates). (G) qRT‐PCR analysis of interleukin (IL) 6 expression after cotransfection with IL6‐AS1 small interfering RNA (siRNA) (siIL6‐AS1‐1) and an IL6‐AS1 overexpression vector in HBF and HFL1 cells (one‐way ANOVA, *n* = 3 biological replicates). (H) ELISA analysis of IL‐6 expression after cotransfection with IL6‐AS1 siRNA (siIL6‐AS1‐1) and an IL6‐AS1 overexpression vector in HBF and HFL1 cells (one‐way ANOVA, *n* = 3 biological replicates). (I and J) qRT‐PCR analysis of IL‐6 expression in knockdown (I) and overexpressing (J) IL6‐AS1 HBF and HFL1 cells in response to stimulation with lipopolysaccharide. Cells were transfected with IL6‐AS1 siRNA (siIL6‐AS1‐1) or control for 48 h and then exposed to LPS (1 μg/ml) for 6 h (one‐way ANOVA, *n* = 4 biological replicates). (K and L) ELISA analysis of IL‐6 expression in knockdown (K) and overexpressing (L) IL6‐AS1 HBF and HFL1 cells in response to stimulation with LPS (one‐way ANOVA, *n* = 4 biological replicates). Error bars represent means ± SD. *p < 0.05, **p < 0.01, and ***p < 0.001

To study the function of IL6‐AS1, two siRNAs specific to IL6‐AS1 (siIL6‐AS1‐1 and siIL6‐AS1‐2) were used to knockdown IL6‐AS1 and overexpress it with a transient transfection vector (OEIL6‐AS1) in both HBF and HFL1 cells (Figure S4A,B). We then measured the expression of inflammation‐related factors and pathways from the coexpression network by knockdown and overexpress IL6‐AS1 as indicated. We found that only the expression of IL‐6 and IL‐8 was regulated by IL6‐AS1 (Figure 2A–D). In addition, IL6‐AS1 knockdown led to a significant decrease in IL‐6 and IL‐8 secretion. In contrast, overexpression of IL6‐AS1 induced a reverse effect (Figure 2E,F). We extracted the protein from the cytoplasm and nucleus and found that AS‐IL6 promoted p50/p65 entering the nucleus to promote inflammation (Figure S4C,D). IL‐6 and IL6‐AS1 are located close to each other on the chromosome, so we selected the IL‐6 gene as a target for IL6‐AS1. Additionally, we obtained similar results for IL‐6 expression, consistent with the transient transfection of IL6‐AS1 siRNA and IL6‐AS1 overexpression vector by using lentivirus‐mediated RNAi or vectors as indicated (Figure S4E–I).

To determine whether cis‐acting regulation is involved^31^, ^32^ and eliminate the locus transcription effect of IL6‐AS1, we measured the expression of its neighboring genes, TOMM7 and STEAP1B, after knockdown and overexpression of IL6‐AS1. We found that IL6‐AS1 did not affect these neighboring genes (Figure S4J), which indicates that IL6‐AS1 specifically regulates IL‐6. Moreover, overexpression of IL6‐AS1 abolished the enhancing effect of IL6‐AS1 knockdown on IL‐6 expression (Figure 2G,H), which shows that the regulatory effects on IL‐6 were attributable to the RNA product of the lncRNA and not to its transcriptional process or locus. Furthermore, we found that IL6‐AS1 amplified IL‐6 expression by using LPS as indicated (Figure 2I–L). Altogether, these findings suggest that IL‐6 is a downstream target of IL6‐AS1 and that this regulation is influenced by the lncRNA product rather than by IL6‐AS1 transcription.

### 3.3 IL6‐AS1 competitively binds to miR‐149‐5p to upregulate IL‐6 in cytoplasm

**FIGURE 3:**
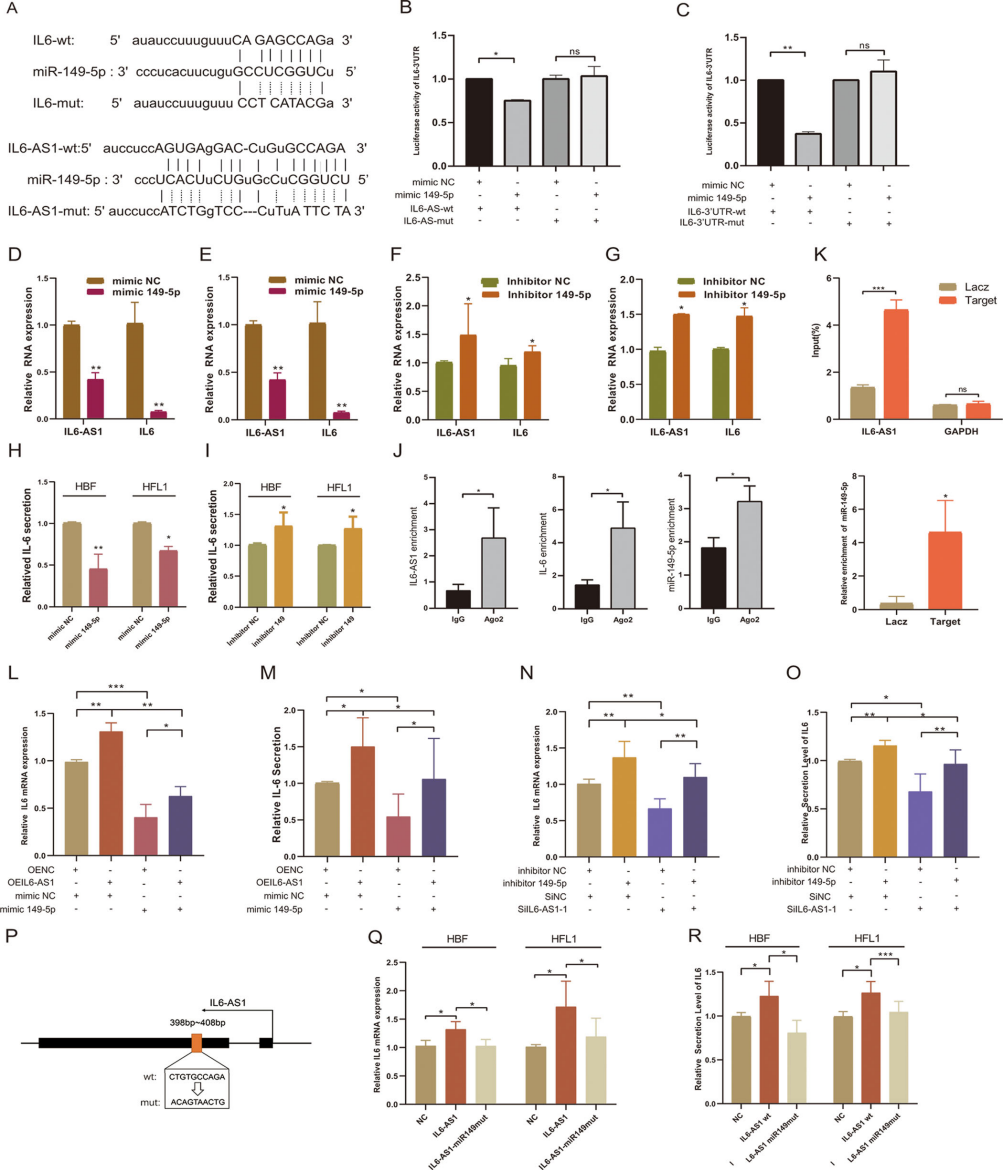
IL6‐AS1 acts as an endogenous sponge by binding to the microRNA miR‐149‐5p to upregulate the expression of IL‐6. (A) Putative miR‐149a‐5p binding sequence of IL6‐AS1 and IL6 3′UTR. Mutation was generated on the IL6‐AS1 and IL6‐3′UTR sequence in the complementary site for the seed region of miR‐149a‐5p. (B and C) Wild‐type IL6‐AS1 (IL6‐AS1‐wt) or mutant IL6‐AS1 (IL6‐AS1‐mut) with the mutation of the miR‐149‐5p binding site were cotransfected with miR‐149‐5p or miR‐NC mimics into 293T cells (B) and wild‐type IL6‐3′UTR (IL6‐3′UTR‐wt) or mutant IL6‐3′UTR (IL6‐3′UTR‐mut) were cotransfected (C). Relative luciferase activity was normalized to Renilla luciferase activity (one‐way ANOVA, *n* = 3 biological replicates). (D and E) qRT‐PCR analysis of IL6‐AS1 and IL‐6 expression in HBF (D) or HFL1 (E) cells transfected with miR‐149‐5p or miR‐NC mimics (paired two‐tailed *t*‐test, *n* = 3 biological replicates). (F and G) qRT‐PCR analysis of IL6‐AS1 and IL‐6 expression in HBF (F) or HFL1 (G) cells transfected with miR‐149‐5p or miR‐NC inhibitors (paired two‐tailed *t*‐test, *n* = 3 biological replicates). (H and I) IL‐6 secretion in HBF and HFL1 cells following transfection with inhibitors of miR‐149‐5p (I) or miR‐149‐5p mimic (H) (paired two‐tailed *t*‐test, *n* = 4 biological replicates). (J) Expression of IL6‐AS1, IL‐6, and miR‐149‐5p in HBF cells following anti‐Ago2 RIP assay (paired two‐tailed *t*‐test, *n* = 4 biological replicates). (K) IL6‐AS1 probe set was used as a ChIRP positive control; GAPDH was detected as a nonspecific control (two‐way ANOVA, *n* = 3 biological replicates). Expression of miR‐149‐5p in HBF cells following ChIRP assay with IL6‐AS1 or GAPDH probe (paired two‐tailed *t*‐test, *n* = 3 biological replicates). (L and M) IL‐6 expression was measured in HBF cells cotransfected with an IL6‐AS1 overexpression vector (OEIL6‐AS1) and miR‐149‐5p mimic by qRT‐PCR (L) and ELISA (M) (one‐way ANOVA, *n* = 4 biological replicates). (N and O) IL‐6 expression was measured in HBF cells cotransfected with an IL6‐AS1 siRNA (SiIL6‐AS1‐1) and miR‐149‐5p inhibitor by qRT‐PCR (N) and ELISA (O) (one‐way ANOVA, *n* = 4 biological replicates). (P) Schematic representation of the mutation sequences of the miR‐149‐5p binding site in IL6‐AS1. (Q and R) IL‐6 expression was measured in HBF and HFL1 cells transfected with wild‐type IL6‐AS1 or mutant IL6‐AS1 of miR‐149‐5p binding site by qRT‐PCR (Q) and ELISA (R) (one‐way ANOVA, *n* = 4 biological replicates). Error bars represent means ± SD. *p < 0.05, **p < 0.01, and ***p < 0.001

Previous studies reported that cytoplasmic lncRNA can act as a molecular sponge or competing endogenous RNA (ceRNA) to achieve functional limitation of miRNA expression to affect biological functions.^33^ Although there was a 67‐bp overlapping region on the chromosome between IL6‐AS1 and IL‐6 (Figure 1F), however, we did not find the mechanism of RNA–RNA duplexing as indicated^34^ (Fig. S5A). To identify ceRNA mechanism and the target miRNAs, we predicted the binding of miRNAs using five bioinformatics databases (miRanda, miRmap,^35^ PicTar,^36^ PITA, and RNA22^37^). A total of nine miRNAs were hypothesized to bind to both IL6‐AS1 and IL‐6 as indicated (Figure S5B). The results of dual‐luciferase reporter assay showed that eight of the nine miRNAs had binding sites in IL‐6 3′UTR, but IL6‐AS1 only had a binding site for miR‐149‐5p, which suggests that miR‐149‐5p may be a target in the ceRNA regulatory network of IL6‐AS1 and IL‐6 (Figure S5C). Furthermore, the expression variations of IL6‐AS1, IL6, and miR‐149‐5p were consistent with the ceRNA network in the RNA‐seq result as indicated (Figure S5D). Furthermore, luciferase activity in cells cotransfected with IL‐6‐3′UTR (wild‐type) and miR‐149‐5p mimics was significantly lower than that in the cells transfected with IL‐6‐3′UTR (mutant) and miR‐149‐5p mimics. Similar results were observed in the IL6‐AS1 (wild‐type and mutant) (Figure 3A–C).

Next, we found that the miRNA exerted regulatory effects in both cell types by using miR‐149‐5p mimic, and the miR‐149‐5p inhibitor reversed that effect as indicated (Figure 3D–I). Consequently, we also found that the expression of miR‐149‐5p was upregulated after knockdown of IL6‐AS1 and downregulated after overexpression as indicated (Figure S5E). To confirm the presence of lncRNA–miRNA–mRNA complex,^38^ we conducted RIP and found that compared with IgG, IL6‐AS1, IL‐6, and miR‐149‐5p were significantly enriched in protein argonaute 2 (AGO2, Figure 3J), which suggests that only IL6‐AS1 possesses miRNA‐related functions. To further validate the mutual direct interaction between IL6‐AS1 and miR‐149‐5p, a ChIRP assay in HBF cells was performed with antisense probe sets against IL6‐AS1 and LacZ; the result indicated that IL6‐AS1 directly binds to miR‐149‐5p (Figure 3K). To verify that IL6‐AS1 regulates IL‐6 through miR‐149‐5p, HBF cells were cotransfected with a miR‐149‐5p mimic and the plasmid for overexpressing IL6‐AS1; the inhibition of IL‐6 by miR‐149‐5p was partially reversed after cotransfection (Figure 3L,M), and vice versa (Figure 3N,O). The similar trends were found in the HFL1 cells treated in the same conditions. (Figure S5F,G). Then, we constructed a mutant IL6‐AS1 vector of the miR‐149‐5p binding site (Figure 3P), and found IL‐6 expression was decreased in the mutant IL6‐AS1 cells compared with the wild‐type IL6‐AS1 cells as indicated (Figure 3Q,R). Altogether, the data suggest that cytoplasmic IL6‐AS1 may act as a ceRNA by binding miR‐149‐5p to upregulate IL‐6 expression.

### 3.4 IL6‐AS1 may function as a transcriptional regulator in nucleus

**FIGURE 4:**
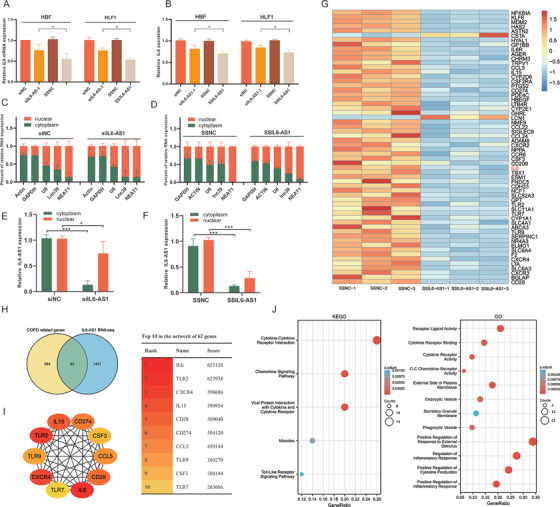
Nuclear IL6‐AS1 regulates inflammation. (A and B) qRT‐PCR and ELISA analysis of the expression (A) and secretion (B) of interleukin (IL) 6 after transfection with SSIL6‐AS1 or siIL6‐AS1‐1 in HBF and HFL1 cells (one‐way ANOVA, *n* = 4 biological replicates). (C and D) Nuclear fractionation analysis and qRT‐PCR analysis of IL6‐AS1 expression in the nucleus and cytoplasm, respectively, after transfection with siIL6‐AS1‐1 (C) or SSIL6‐AS1 (D) (two‐way ANOVA, *n* = 3 biological replicates). (E and F) IL6‐AS1 RNA fold‐change in the nucleus and cytoplasm after transfection with siIL6‐AS1‐1 or SSIL6‐AS1 (*n* = 3 biological replicates). (G) Heatmap representing 62 genes that intersect between IL6‐AS1 RNA‐seq DEGs and COPD‐related genes. Rows represent genes and columns represent treated samples. Blue, downregulation; red, upregulation. (H) Intersection of the Venn diagram shows the overlapping genes between both comparisons DEGs in IL6‐AS1 RNA‐seq and COPD‐related genes. (I) Schematic representation of the top 10 genes in the PPI network of 62 genes. The higher the score, the more important the gene. (J) Schematic representation of the KEGG and GO analysis of 62 genes. Error bars represent means ± SD. *p < 0.05 and ***p < 0.001

We found that IL6‐AS1 is also located in the nucleus and at higher levels than in the cytoplasm. However, the ceRNA mechanism does not fully account for the regulatory effect of IL6‐AS1 on IL‐6. lncRNAs in the nucleus have been suggested to be involved in gene regulation by influencing transcription.^15^ Thus, we speculated that IL6‐AS1 exerts a similar regulatory effect in the nucleus. We used a smart silencer specific to IL6‐AS1 (SS‐IL6‐AS1) that was composed of three siRNAs and three antisense oligonucleotides. The transfection efficiency was higher than that of siRNA in both HBF and HFL1 cells (Figure S6A). Similarly, we observed that the expression and secretion of IL‐6 were significantly lower after transfection with SS‐IL6‐AS1 than with siIL6‐AS1 (Figure 4A,B). Additionally, we extracted nucleic and cytoplasmic RNA from HBF cells after separate transfections with SS‐IL6‐AS1 and siIL6‐AS1‐1, then measured the change in IL6‐AS1 expression by qRT‐PCR (Figure 4C,D). Both SSIL6‐AS1 and siIL6‐AS1‐1 affected IL6‐AS1 in the cytoplasm, but only SS‐IL6‐AS1 reduced the expression of IL6‐AS1 in the nucleus (Figure 4E,F).

CeRNAs may affect specific genes, but the mechanism of lncRNA action in the nucleus is related to transcriptional regulation, which may affect a series of related genes. To determine the functional effect of IL6‐AS1 in the nucleus, we performed RNA sequencing after transfection of HBF cells with SSIL6‐AS1. In total, 1494 RNAs were differentially expressed, and KEGG and GO enrichment analysis showed that these differentially expressed genes (DEGs) were enriched for cytokine–cytokine receptor interaction (Figure S6B). GSEA yielded similar conclusions (Figure S6C). We then identified 24 genes, including IL‐6, that overlapped between the RNA‐seq and lncRNA‐mRNA coexpression network genes (Figure S6D and Table S2). In addition, to investigate whether the DEGs might be involved in regulating COPD pathogenesis, we determined the genes that overlapped between RNA‐seq‐ and COPD‐related genes (genes were selected based on a score ≥2 from GeneCards) and 62 genes were included (Figure 4G,H and Table S3). PPI networks were constructed using these 62 genes using the STRING database^39^ (Figure S6E) and the hub genes were identified using cytoHubba in Cytoscape. From this analysis, the IL‐6 score was the highest and IL‐6 was the key gene in the PPI network (Figure 4I). In addition, we performed KEGG pathway and GO enrichment analysis on the intersection genes and found them to be associated with inflammation (Figure 4J), which indicated that IL6‐AS1 plays an important role in abnormal inflammatory responses in COPD. Overall, these findings indicate that IL6‐AS1 in the nucleus might play a role in regulating IL‐6, and that IL‐6 is not just an IL6‐AS1 target gene but also is the core gene in the IL6‐AS1 regulatory network.

### 3.5 IL6‐AS1 interacts with EBF1 to regulate IL‐6 expression by affecting binding of EBF1 on IL‐6 promoter

**FIGURE 5:**
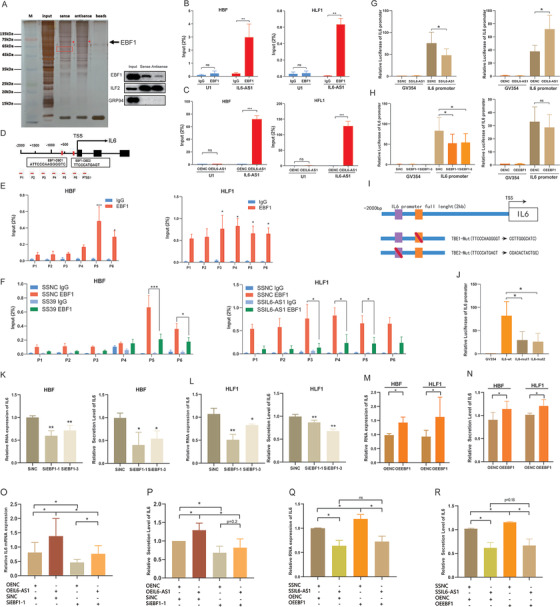
IL6‐AS1 interacts with EBF1 to regulate IL‐6 expression by affecting the binding of EBF1 to the IL‐6 promoter. (A) RNA pull‐down assay using IL6‐AS1 sense and antisense RNAs in HBF cells, followed by silver staining. Black arrow indicates EBF1. The interaction between IL6‐AS1 and EBF1 was confirmed by Western blotting with the extract of the RNA pull‐down assay. ILF2 and GPR94 were also detected. (B) RIP‐qPCR analysis using an anti‐EBF1 antibody showed that IL6‐AS1 interacts with endogenous EBF1 in HBF and HFL1 cells. U1 was used as the negative control (two‐way ANOVA, *n* = 3 biological replicates). (C) RIP‐qPCR analysis with an anti‐EBF1 antibody was conducted after overexpression of IL6‐AS1 in HBF and HFL1 cells (two‐way ANOVA, *n* = 3 biological replicates). (D) Schematic representation of potential EBF1 binding sites on the IL‐6 promoter. As promoters considered to be located 2000 bp upstream of the transcription start site (TSS), we designed six pairs of chromatin immunoprecipitation (ChIP)‐qPCR primers to cover the IL‐6 promoter region, with the fifth and sixth primers containing two EBF1 binding sites. (E) ChIP‐PCR analysis of EBF1 occupancy on the IL‐6 promoter in HBF and HLF cells. IgG was used as the negative control (two‐way ANOVA, *n* = 3 biological replicates). (F) ChIP‐PCR analysis of EBF1 occupancy on the IL‐6 promoter after transfection with an IL6‐AS1 silencer (SSIL6‐AS1) in HBF and HLF cells (two‐way ANOVA, *n* = 3 biological replicates). (G) Luciferase activity in the IL‐6 promoter following cotransfection of the IL6‐AS1 silencer (SSIL6‐AS1) or IL6‐AS1 overexpression vector (two‐way ANOVA, *n* = 4 biological replicates). (H) Luciferase activity in the IL‐6 promoter following cotransfection of an EBF1 small interfering RNA (siRNA) (siEBF1‐1) and EBF1 overexpression vector (two‐way ANOVA, *n* = 4 biological replicates). (I) Schematic representation of the two mutation sequences of potential EBF1 binding sites on the IL‐6 promoter. (J) Luciferase activity in the IL‐6 promoter following transfection with a reporter containing wild‐type or mutant IL‐6 promoter (one‐way ANOVA, *n* = 4 biological replicates). (K and L) qRT‐PCR and ELISA analysis of IL‐6 expression after transfection with two EBF1 siRNAs in HBF cells (K) or HLF cells (L) (one‐way ANOVA, *n* = 4 biological replicates). (M and N) qRT‐PCR and ELISA analysis of IL‐6 expression after overexpression of EBF1 in HBF cells (M) or HLF cells (N) (one‐way ANOVA, *n* = 4 biological replicates). (O and P) Expression of IL‐6 in HBF cells following cotransfection with IL6‐AS1 overexpression vector and EBF1 siRNA (SiEBF1‐1), determined by qRT‐PCR (O) and ELISA (P) (one‐way ANOVA, *n* = 5 biological replicates). (Q and R) Expression of IL‐6 in HBF cells following cotransfection with an IL6‐AS1 Smart Silencer (SSIL6‐AS1) and EBF1 overexpression vector, determined by qRT‐PCR (Q) and ELISA (R) (one‐way ANOVA, *n* = 5 biological replicates). Error bars represent means ± SD. *p < 0.05, **p < 0.01, and ***p < 0.001

lncRNAs regulate gene transcription by binding proteins and recruiting transcriptional programs.^34^, ^40^, ^41^ We identified EBF1, a transcription factor located in a specific protein band by using RNA pull‐down and mass spectrometry, which may be a target protein of IL6‐AS1. EBF1 was detected in the IL6‐AS1 pull‐down complex but not in the control sample pulled down by an IL6‐AS1 antisense transcript, ILF2 was not differently expressed and GRP94 has not been detected as indicated (Figure 5A). We predicted the interaction between IL6‐AS1 and EBF1 by using RNAInter^42^ and RPISEquation (Figure S7A,B). Then, RIP results showed that compared with the negative control (IgG), there was a notable interaction between IL6‐AS1 and EBF1 (Figure 5B). Similarly, IL6‐AS1 overexpression increased the IL6‐AS1 enrichment in cells treated with an EBF1 antibody as indicated (Figure 5C). These indicated that IL6‐AS1 interacts with EBF1. According to the JASPAR database^43^ through the UCSC Genome Browser, we found that the IL‐6 promoter contains a binding site for EBF1 (Figure S7C). In parallel, we utilized the ENCODE database^44^ (http://genome.ucsc.edu/ENCODE/) and retrieved two ChIP‐seq data of EBF1 binding sites^45^, ^46^ (GSM803386 and GSM935375); the results showed that there are two peaks of EBF1 binding at the IL‐6 promoter (Figure S7D, Table S2). Meanwhile, we discovered most of IL6‐AS1‐related genes contain chromatin‐binding sites of EBF1 (Figure S7E), which indicated there were similar effects between IL6‐AS1 and EBF1.

**FIGURE 6:**
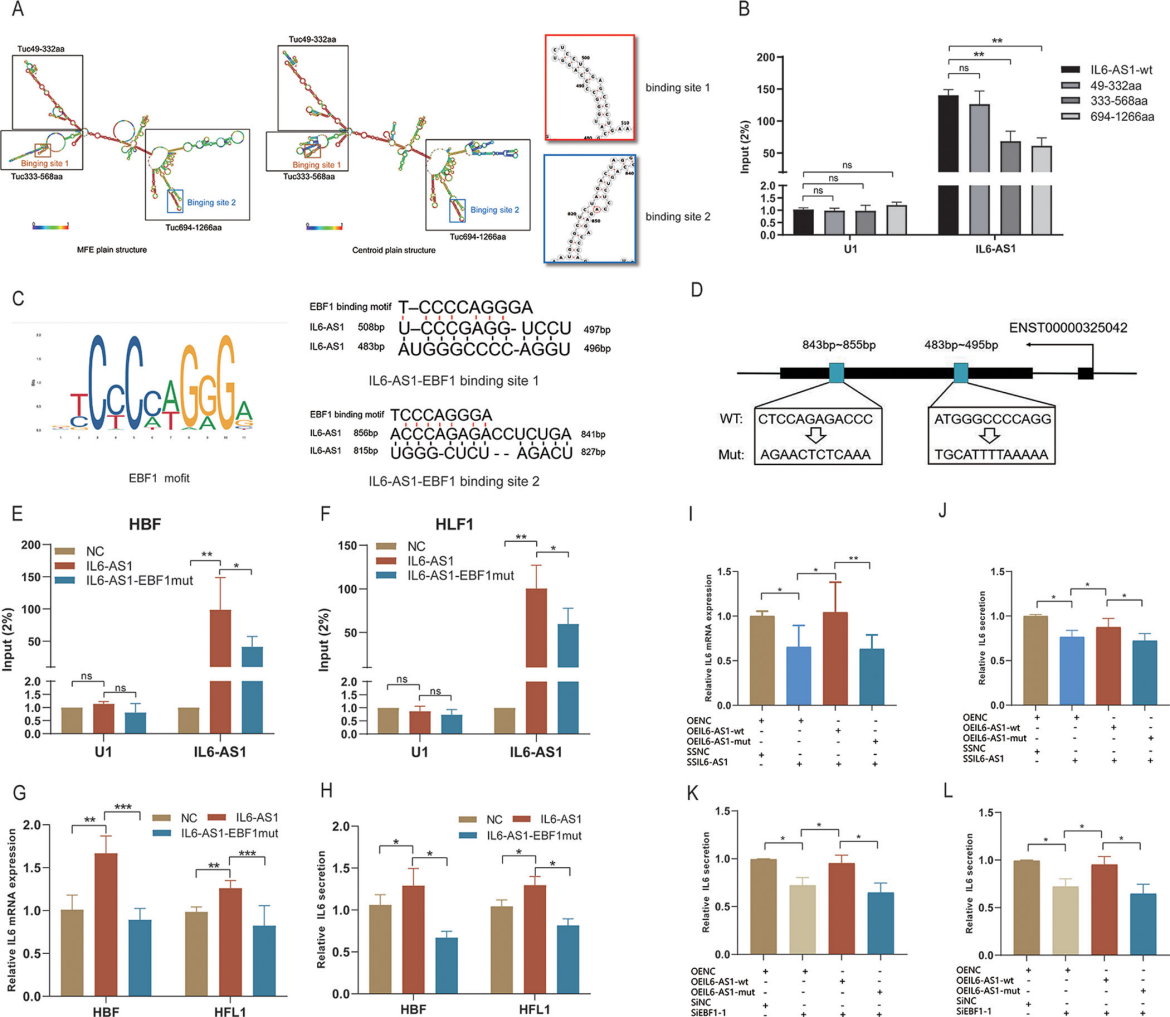
Two hairpin structures of IL6‐AS1 can bind to the EBF1 protein. (A) Two computational methods (centroid plain structure and minimum free energy plain structure) were used to compute secondary structures of IL6‐AS1 using the RNAfold database (http://rna.tbi.univie.ac.at/cgi‐bin/RNAWebSuite/RNAfold.cgi). The sequence regions in red are the stable regions in the IL6‐AS1 secondary structure and the blue sequence regions are the unstable regions. The hairpin structures in the red and blue boxes are the potential binding sites of the EBF1 transcription factor. The structures in the black boxes are the highly conserved stem‐loops. (B) RIP‐qPCR analysis with an anti‐EBF1 antibody after transfection with wild‐type or truncated IL6‐AS1 (49‐332aa, 333‐568aa, 694‐1266aa) in HBF cells (two‐way ANOVA, *n* = 3 biological replicates). (C and D) Schematic representation of the EBF1 binding motif (C) and the two mutation sequences of potential EBF1 binding sites (D) in IL6‐AS1. (E and F) RIP‐qPCR analysis with an anti‐EBF1 antibody after transfection with wild‐type or mutant IL6‐AS1 in HBF cells (E) and HFL1 cells (F) (two‐way ANOVA, *n* = 4 biological replicates). (G and H) qRT‐PCR (G) and ELISA (H) analysis of IL‐6 expression after transfection with wild‐type or mutant IL6‐AS1 in HBF cells and HFL1 cells (one‐way ANOVA, *n* = 3 biological replicates). (I and J) HBF cells were cotransfected with wild‐type or mutant IL6‐AS1 and SSIL6‐AS1 and IL‐6 expression was determined by qRT‐PCR (I) and ELISA (J) (one‐way ANOVA, *n* = 4 biological replicates). (K and L) HBF cells were cotransfected with wild‐type or mutant IL6‐AS1 and siEBF1‐1 and IL‐6 expression was determined by qRT‐PCR (K) and ELISA (L) (one‐way ANOVA, *n* = 4 biological replicates). Error bars represent means ± SD. *p < 0.05, **p < 0.01, and ***p < 0.001

In order to verify the accuracy of above data in online database, ChIP assay was performed and the results showed that EBF1 bound directly to the IL‐6 promoter region by ChIP (Figure 5D,E). We further found knockdown of IL6‐AS1 decreased the binding efficiency of EBF1 to the IL‐6 promoter (Figure 5F) and nuclear translocation of EBF1 as indicated (Figure S8A).

To confirm the effects of IL6‐AS1 on the IL‐6 promoter, we found knockdown IL6‐AS1 decreased the luciferase activity of the IL‐6 promoter and overexpression reversed that effect (Figure 5G). Next, the results of knockdown and overexpression of EBF1 were almost identical to those of knockdown and overexpression of IL6‐AS1 by using two siRNAs (siEBF1‐1 and siEBF1‐3) or an EBF1 overexpression vector (Figure 5H and Figure S8B,C). Furthermore, we found that the mutant of EBF1 binding sites decreased basal luciferase activity of IL‐6 promoter (Figure 5I,J). These data suggest that IL6‐AS1 regulates IL‐6 promoter activity through EBF1 binding to the IL‐6 promoter site.

However, we found no published reports describing the regulatory relationship between EBF1 and the IL‐6 gene. The results showed that knockdown of EBF1 decreased IL‐6 expression (Figure 5K,L) but had no significant influence on IL6‐AS1 expression (Figure S9A,B), and overexpression of EBF1 induced a reverse effect (Figure 5M,N and Figure S8C). These results suggested that IL‐6 is regulated by EBF1 and the effect of EBF1 is similar to the function of IL6‐AS1. Additionally, knockdown or overexpression of IL6‐AS1 notably affected EBF1 protein levels (Figure S9D,E) but not mRNA levels (Figure S9F,G). Thus, we hypothesized that IL6‐AS1 affects the stability and degradation of the EBF1 protein by direct binding. To verify that EBF1 acts as the intermediate regulator of IL6‐AS1‐induced IL‐6 expression, we performed a series of rescue experiments as indicated, both overexpression and knockdown of IL6‐AS1 in cells partly recovered the EBF1 interference‐induced expression of IL‐6 as indicated (Figure 5O–R and Figure S9H,I). IL6‐AS1 therefore regulates IL‐6 expression by recruiting EBF1 to the IL‐6 promoter region and affecting promoter activity.

### 3.6 EBF1 may directly bind RNA hairpin structures of IL6‐AS1

lncRNAs are known to form complex secondary structures via base pairing, and the function of lncRNAs has been reported to be mainly determined by the secondary structures because the proteins bind to target RNA hairpin structures.^16^ We used both centroid plain structure and minimum free energy plain structure to predict RNA secondary structures using RNAfold,^47^ and the structural model showed that there are three stem‐loops (49‐332aa, 333‐568aa and 694‐1266aa) in IL6‐AS1, then we separately constructed three different stem‐loop truncations of IL6‐AS1 as indicated (Figure 6). RIP results showed that compared with the IL6‐AS‐wt, tuc333‐568aa and tuc694‐1266aa of IL6‐AS1 significantly decreased EBF1 enrichment, but tuc49‐332aa had no significant influence (Figure 6). In 333‐568aa and 694‐1266aa stem‐loops, we further found two predicted secondary structures with high conservatism directly binding to EBF1 according to the EBF1 binding motif (Figure 6). To characterize the interaction between specific hairpin structures and EBF1, we mutated the hairpin structure (Figure 6), and the IL6‐AS1 mutation appeared to reduce the degree of enrichment of IL6‐AS1 as indicated (Figure 6). Furthermore, we found mutant IL6‐AS1 exerted a significant callback effect on wild‐type IL6‐AS1 and decreased IL‐6 expression in HBF and HFL1 cells (Figure 6). In addition, the mutant IL6‐AS1 partly decreased the wild‐type‐induced rescue effects of IL‐6 expression as indicated (Figure 6). Similarly, HBF cells were cotransfected with wild‐type or mutant IL6‐AS1 and siEBF1‐1, and similar results were obtained (Figure 6). In summary, IL6‐AS1 binds directly to EBF1 by its specific hairpin loop structure, and the IL6‐AS1/EBF1 complex has functional significance.

**FIGURE 7:**
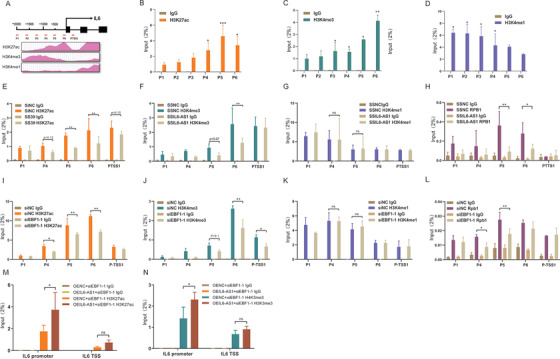
IL6‐AS1 promotes the modification of the histones H3K4me3 and H3K27ac on the interleukin (IL) 6 promoter, probably through EBF1. (A) Schematic representation of the putative modification markers, H3K27ac, H3K4me1, and H3K4me3 upstream of IL‐6 from the ENCODE database (https://genome.ucsc.edu/ENCODE/). (B–D) ChIP‐PCR analysis of H3K27ac (B), H3K4me1 (C), and H3K4me3 (D) on the IL‐6 promoter in HBF cells (two‐way ANOVA, *n* = 3 biological replicates). (E–H) HBF cells were transfected with SSIL6‐AS1 and assessed for H3K27ac (E), H3K4me3 (F), H3K4me1 (G) and RNA polymerase II (H) on the IL‐6 promoter by ChIP‐qPCR analysis (two‐way ANOVA, *n* = 3 biological replicates). (I–L) HBF cells were transfected with siEBF1‐1 and assessed for H3K27ac (I), H3K4me1 (J), H3K4me3 (K), and RNA polymerase II (L) on the IL‐6 promoter by chromatin immunoprecipitation‐qPCR analysis (two‐way ANOVA, *n* = 3 biological replicates). (M and N) HBF cells were cotransfected with siEBF1‐1 and IL6‐AS1 overexpression vector and assessed for H3K27ac (M) and H3K4me3 (N) on the IL‐6 promoter by ChIP‐qPCR analysis (two‐way ANOVA, *n* = 3 biological replicates). Error bars represent means ± SD. *p < 0.05, **p < 0.01, and ***p < 0.001

### 3.7 IL6‐AS1 may regulate histone modification on IL‐6 promoter through EBF1

The binding of EBF1 has been reported to modulate histone modification in chromatin.^48^ We speculated that IL6‐AS1 may affect histone modifications at IL‐6 promoter by regulating EBF1 binding. We identified the putative histone modification markers, H3K27ac, H3K4me1, and H3K4me3, upstream of IL‐6 in IMR‐90 cells by ChIP‐seq using the ENCODE whole‐genome database (Figure 7A). Using the antibodies for H3K27ac, H3K4me1, and H3K4me3 and RNA polymerase II, we found that the extent of each histone modification at the IL‐6 promoter was consistent with the ChIP‐seq data provided by ENCODE in HBF cells (Figure 7B–D). We measured the effect of IL6‐AS1 on histone modification at the IL‐6 promoter and found knockdown of IL6‐AS1 resulted in the reduction of H3K4me3, H3K27ac, and RNA polymerase II but not H3K4me1 on the IL‐6 promoter in HBF cells (Figure 7E–H). We found that knockdown of EBF1 reduced H3K4me3, H3K27ac, and RNA polymerase II but not H3K4me1, in general agreement with a previous report^48^ (Figure 7I–L). In addition, overexpression of IL6‐AS1 partly recovered the EBF1 interference‐induced histone modication of IL‐6 promoter as indicated (Figure 7M,N). Collectively, the experimental results and the IL6‐AS1/EBF1 complex demonstrate that IL6‐AS1 may regulate histone modification on the IL‐6 promoter by affecting EBF1 binding, but the exact mechanism of action still requires study.

### 3.8 Two mechanisms of IL6‐AS1 action synergize in regulating IL‐6 expression

**FIGURE 8:**
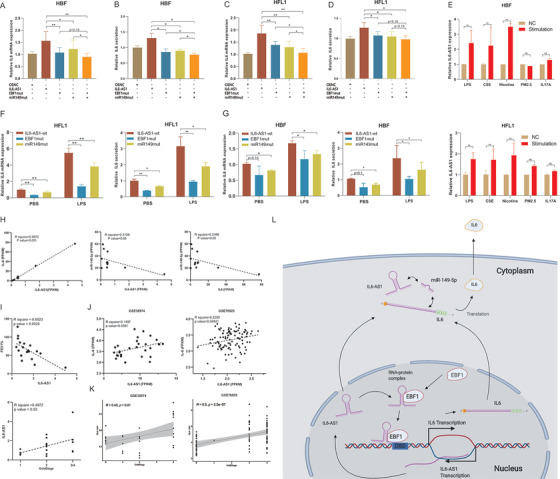
Two mechanisms that synergize to promote interleukin (IL) 6 expression. (A and B) qRT‐PCR analysis of IL‐6 expression after cotransfection of HBF cells (A) and HFL1 cells (B) with wild‐type IL6‐AS1 or EBF1‐mutant IL6‐AS1 and miRNA‐mutant IL6‐AS1 (one‐way ANOVA, *n* = 5 biological replicates). (C and D) ELISA analysis of IL‐6 secretion after cotransfection of HBF cells (C) and HFL1 cells (D) with wild‐type IL6‐AS1 or EBF1‐mutant IL6‐AS1 and miRNA‐mutant IL6‐AS1 (one‐way ANOVA, *n* = 5 biological replicates). (E) HBF and HFL1 cells were exposed to LPS (500 ng/ml), cigarette smoke extract (CSE, 0.015%), PM_2.5_ (2 μg/ml), IL17A (200 ng/ml), or nicotine (10 μM) for 24 h. qRT‐PCR analysis of IL6‐AS1 expression (two‐way ANOVA, *n* = 3 biological replicates). (F and G) qRT‐PCR and ELISA analysis of IL‐6 expression in wild‐type IL6‐AS1 cells, EBF1‐mutant IL6‐AS1 or miRNA‐mutant IL6‐AS1 cells after exposure to lipopolysaccharide (500 ng/ml) for 24 h in HFL1 (F) and HBF cells (G) (two‐way ANOVA, *n* = 3 biological replicates). (H) Correlation analysis of gene expression between IL6‐AS1/IL‐6, IL6‐AS1/miR‐149‐5p, and IL6/miR‐149‐5p from RNA‐seq results. (I) Correlation analysis between the expression of IL6‐AS1 and FEV1% in verified samples; correlation analysis between the expression of IL6‐AS1 and GOLD stage in verified samples. (J) Correlation analysis of gene expression between IL6‐AS1/IL‐6 in GSE38974 and GSE76925. (K) Correlation analysis between the expression of IL6‐AS1 and GOLD stage in GSE38974 and GSE76925. (L) Overview of the involvement of IL6‐AS1 in chronic obstructive pulmonary disease (COPD). Schematic representation of the mechanisms by which IL6‐AS1 regulates IL‐6 expression: promoting transcription and affecting histone modification by direct binding with EBF1 in the nucleus, and stabilizing IL‐6 mRNA by acting as a competing endogenous RNA (ceRNA) for miR‐149‐5p in the cytoplasm. Error bars represent the mean ± SD. *p < 0.05 and **p < 0.01

A few studies have reported that lncRNAs act as endogenous sponges by competitively binding to miRNAs and also as RNA scaffolds to recruit proteins.^49^ To assess whether these mechanisms act synergistically to regulate IL‐6 expression, in experiments we found the upregulation of IL‐6 expression by wild‐type IL6‐AS1 was partly attenuated by the mutation of either individual EBF1 site or miR‐149‐5p site, and the attenuation effect by the simultaneous mutation of both sites was almost completely prevented as indicated (Figure 8A–D). In addition, to explore whether there is a direct cross‐talk between IL6‐AS1/miR‐149‐5p/IL6, and IL6‐AS1/EBF1/IL6 pathways, we silenced EBF1 and measured whether miR‐149‐5p expression was changed, and results indicated that EBF1 had no significant effect on miR‐149‐5p. Meanwhile, knockdown miR‐149‐5p had no significant effect on EBF1 expression (Figure 8E,F). These results indicated that there was no direct interaction between IL6‐AS1/miR‐149‐5p/IL6 and IL6‐AS1/EBF1/IL6 pathways, and they are relatively independent. There are many factors that affect chronic airway inflammation, such as cigarette smoke extract (CSE), LPS, particulate matter (PM_2.5_), and some inflammatory factors.^6^ Thus, HBF and HFL1 cells were stimulated with LPS, CSE, PM_2.5_, IL17A, and nicotine to construct inflammation model in vitro, in agreement with literature.^50^, ^51^, ^52^ LPS, CSE, and nicotine significantly promoted the expression of IL6‐AS1 and IL‐6, while the expression of miR‐149‐5p was reduced. These results were consistent with the ceRNA pattern, but IL17A and PM_2.5_ had no significant influence on HBF and HFL1 cells (Figure 8E and Figure S10C,D). Meanwhile, both IL6‐AS1 with a mutant EBF1 binding site and IL6‐AS1 with a mutant miR‐149‐5p binding site can partially reduce the increased expression of IL‐6 by LPS, indicating that these binding sites might be the key players in the development of airway inflammation (Figure 8F,G). Finally, we conducted correlation analysis for IL6‐AS1. Our RNA‐seq results showed that IL6‐AS1 was significantly positively correlated with IL‐6, and miR‐149‐5p was significantly negatively correlated with IL‐6 and IL6‐AS1 (Figure 8H), the gene expression changes in COPD were consistent with the regulatory mechanisms of ceRNA, and mutual positive correlation was demonstrated by IL6‐AS1/IL‐6, IL6‐AS1/EBF1, and IL‐6/EBF1 in lung tissue. Screening the online database, gene expression profiling interactive analysis (GEPIA)2^53^ (http://gepia2.cancer‐pku.cn/), also indicated that the regulatory mechanism for IL‐6 may exist in vivo. We discovered that the expression of IL6‐AS1 is negatively related with FEV1% and positively related with GOLD (Global Initiative for Chronic Obstructive Lung Disease) stage among the clinical samples in Figure 1C (Figure 8I). We also discovered that IL6‐AS1 and IL‐6 were correlated in GSE38974 and GSE76925 (Figure 8J), and found that IL6‐AS1 expression was positively related to GOLD grade (Figure 8K). This indicated that IL6‐AS1 expression is closely related to the severity of COPD and may serve as a biomarker and therapeutic target. Together, these results show that these two regulatory mechanisms can function concurrently in vitro. Figure 8L shows a schematic diagram of the different IL6‐AS1 mechanisms in the cytoplasm and nucleus.

## 4 DISCUSSION

Multiple inflammatory factors contribute to COPD, leading to frequent injury and repair that result in airway structural changes and remodeling of the airway, although chronic inflammation may be central to the pathogenesis of COPD,^6^, ^54^, ^55^ the precise mechanism of this process is unclear. In the present study, we identified a lncRNA, IL6‐AS1, that is overexpressed in COPD and modulates the expression of the inflammatory cytokine IL‐6 in fibroblasts. Mechanistically, IL6‐AS1 acts as an endogenous sponge by competitively binding to miR‐149‐5p to maintain the stability of IL‐6 mRNA. IL6‐AS1 also regulates IL‐6 promoter activity by recruiting the transcription factor EBF1 in the nucleus, thereby increasing the levels of H3K4me3 and H3K27ac. IL6‐AS1‐mediated regulation involves epigenetic control of IL‐6 expression, as well as transcriptional and posttranscriptional (transcript stability) mechanisms. In this study, we identified a potential regulatory mechanism of IL‐6 secretion from structural cells that accounts for IL‐6 accumulation in local airway fibroblasts, which may relate to airway inflammation. IL6‐AS1 expression is correlated with IL‐6 levels and the effect is direct rather than secondary, indicating that this is an early event in the development of COPD. Our findings suggest novel insight into the lncRNA‐mediated modulation of airway inflammation that involves the development and progression of COPD. Although we did not research the downstream effects of IL‐6, previous studies report that inflammation invariably precedes airway wall fibrosis, aging and other pathological changes in fibroblast during COPD, which indicated that fibroblast‐related inflammation is important.^56^ IL‐6 is a pleiotropic cytokine involved in mediating the progression of a number of lung diseases, including COPD. In the immune system, IL‐6 is involved in the differentiation of dendritic cells^57^ and modulates the balance between Th1 and Th2 effector functions.^58^ IL‐6 can also regulate aspects of the CD4 T cell‐mediated response, such as functional aspects of non‐immune cells including epithelial cells and fibroblasts.^59^ Elevated levels of IL‐6 in induced sputum from COPD patients show the inverse correlation between IL‐6 levels and lung function.^60^ Thus, local increases in IL‐6 signaling are involved in inflammation and tissue structure damage. Local concentrations of inflammatory factors such as IL‐6 in structural cells may affect the regulation of immune cells.

EBF1 is highly conserved and is a key factor of B‐cell growth and differentiation in B lymphocytes.^61^ EBF1 enhances the transcription of target genes, and knockdown of EBF1 reduces expression of genes related to inflammatory signaling and secretion of inflammatory cytokines in adipogenesis.^62^, ^63^, ^64^ EBF1 binding induces changes in chromatin structure, including histone modifications, in B lymphopoiesis.^48^, ^65^ To the best of our knowledge, EBF1 preferentially binds to RNA.^66^ In the present study, we found that there are similar functional effects between IL6‐AS1 and EBF1. Potential binding sites for EBF1 were found in the promoter region of the IL‐6 gene, and we demonstrated that EBF1 binds to the IL‐6 promoter site and can promote IL‐6 expression by activating the promoter and facilitating histone modification. Although the expression of EBF1 is stable, we speculate that EBF1 binding is involved and requires IL6‐AS1 as a scaffold, which underscores the importance of EBF1 recruitment by IL6‐AS1.

Although lncRNAs have been reported to function in both the nucleus and cytoplasm, few studies have reported synergy between the two locales. Cytoplasmic lncRNAs mainly act as an endogenous sponge by competitively binding to target miRNAs to upregulate target mRNA,^67^ while nuclear lncRNAs promote the binding of transcription factors to the DNA‐binding domain and protein stabilization,^68^ thereby regulating transcription.^69^ We found that IL6‐AS1 has regulatory functions in both the nucleus and cytoplasm and elucidated a ceRNA regulatory network of IL6‐AS1/miR‐149‐5p/IL‐6 in the pathogenesis of COPD. Kong et al. showed that miR‐149‐5p binds to and stabilizes IL‐6 mRNA in nasopharyngeal carcinoma cells,^70^ while Wang et al. found that AS‐IL6 induces enrichment of H3K27ac at the IL‐6 promoter in glioma cells,^71^ which is similar to what we found. However, we also found that EBF1 may be the key regulatory factor in histone modification and transcription. IL6‐AS1 can bind directly to EBF1, which stabilizes the EBF1 protein and influences its ability to bind to the IL‐6 promoter. However, the molecular mechanisms by which the IL6‐AS1/EBF1 complex recognizes the IL‐6 promoter region and upregulates IL‐6 expression require further study. In addition, EBF1 has not been previously reported in fibroblasts and we only focused on its relationship with IL‐6; whether EBF1 can regulate other inflammatory factors remains to be determined. Finally, we mutated in IL6‐AS1 for miR‐149‐5p and EBF1 individually and simultaneously to confirm the synergistic effect, which suggests that IL6‐AS1 may be a causative agent in the development of COPD. Sequence conservation of lncRNAs is often quite poor between species,^72^ and was found for IL6‐AS1. IL6‐AS1 has no orthologous gene in mouse or rat, so the function of IL6‐AS1 in vivo may be difficult to determine, which is a limitation of the present study.

In summary, our study provides solid evidence supporting the hypothesis that IL6‐AS1 is functionally relevant to inflammation in COPD by promoting IL‐6 expression and outlines a novel mechanism of IL6‐AS1 regulation of COPD progression via EBF1 and IL‐6. IL6‐AS1 is, therefore, a promising molecular target of COPD therapy.

## CONFLICT OF INTEREST

The authors declare that there is no conflict of interest.

## AUTHOR CONTRIBUTIONS

Erkang Yi, Pixin Ran, and Bing Li conceived and designed the experiments. Erkang Yi, Jiahuan Zhang, Mengning Zheng, Yi Zhang, Chunxiao Liang, Binwei Hao, Wei Hong, Biting Lin, Jinding Pu, Zhiwei Lin and Peiyu Huang performed the experiments. Erkang Yi wrote the manuscript and analyzed the data. Pixin Ran, Yumin Zhou, and Bing Li were responsible for research supervision and funding acquisition.

## Supporting information

Supporting Information

Supporting Information

Supporting Information

Supporting Information

Supporting Information

Supporting Information

Supporting Information

Supporting Information

Supporting Information

Supporting Information

Supporting Information

## Data Availability

All the data and materials are available from the corresponding author upon reasonable request.
