# Supplementary figures and images for: Long noncoding RNA IL6‐AS1 is highly expressed in chronic obstructive pulmonary disease and is associated with interleukin 6 by targeting miR‐149‐5p and early B‐cell factor 1
Source: Clin Transl Med. 2021 Jul 19;11(7):e479. doi: 10.1002/ctm2.479 (PMC8288003; doi:10.1002/ctm2.479)

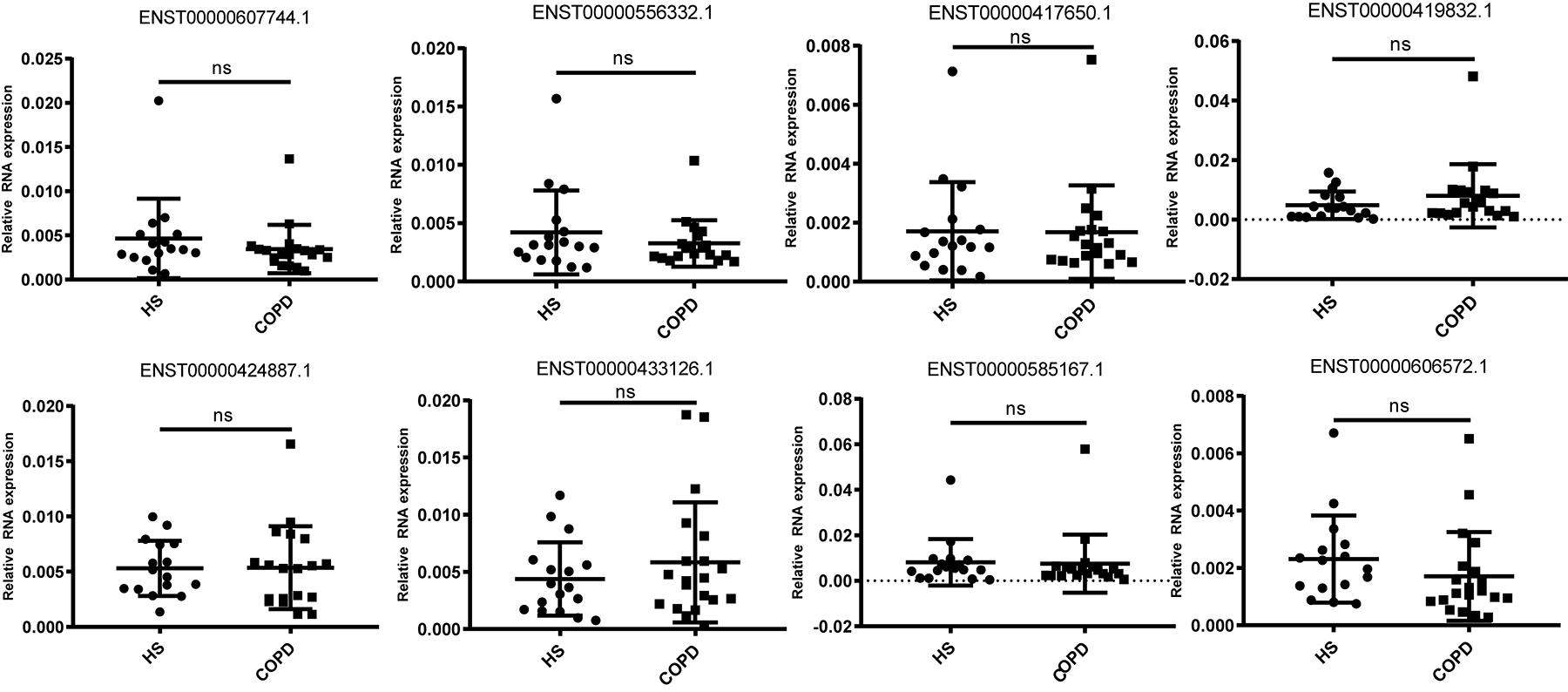

Supplement: Supplementary file 1 — Supporting Information [file CTM2-11-e479-s003.tif]

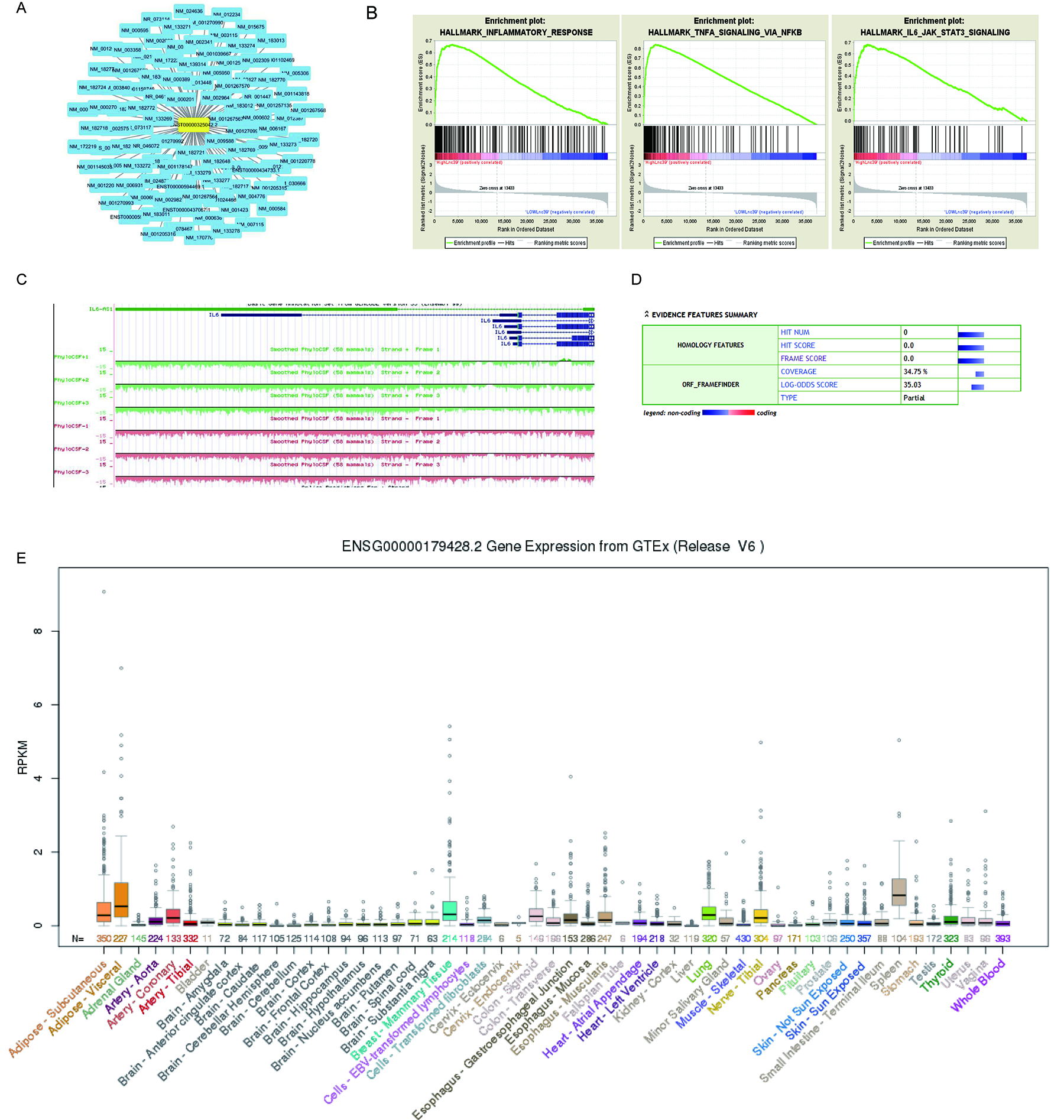

Supplement: Supplementary file 2 — Supporting Information [file CTM2-11-e479-s007.tif]

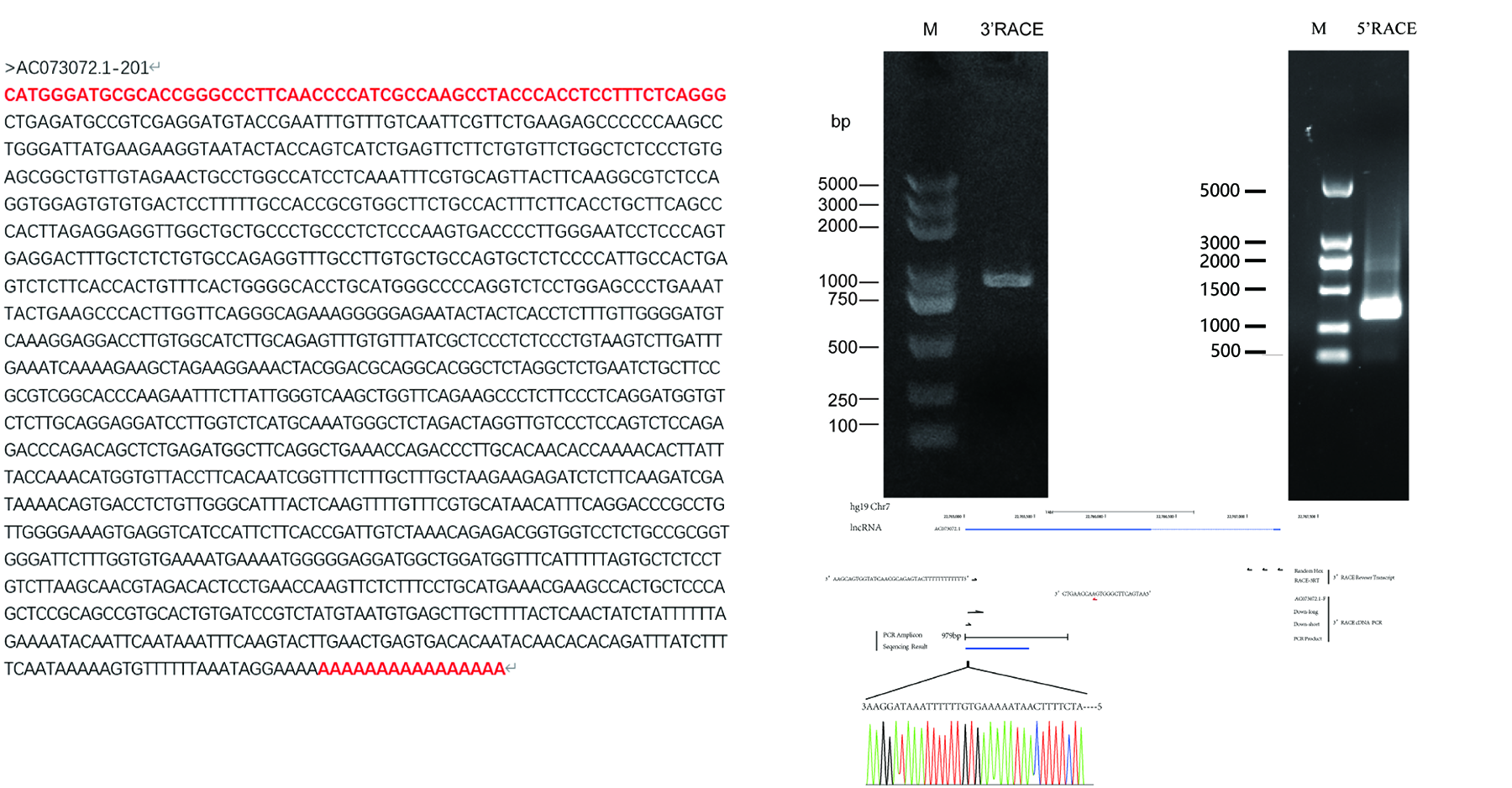

Supplement: Supplementary file 3 — Supporting Information [file CTM2-11-e479-s005.tif]

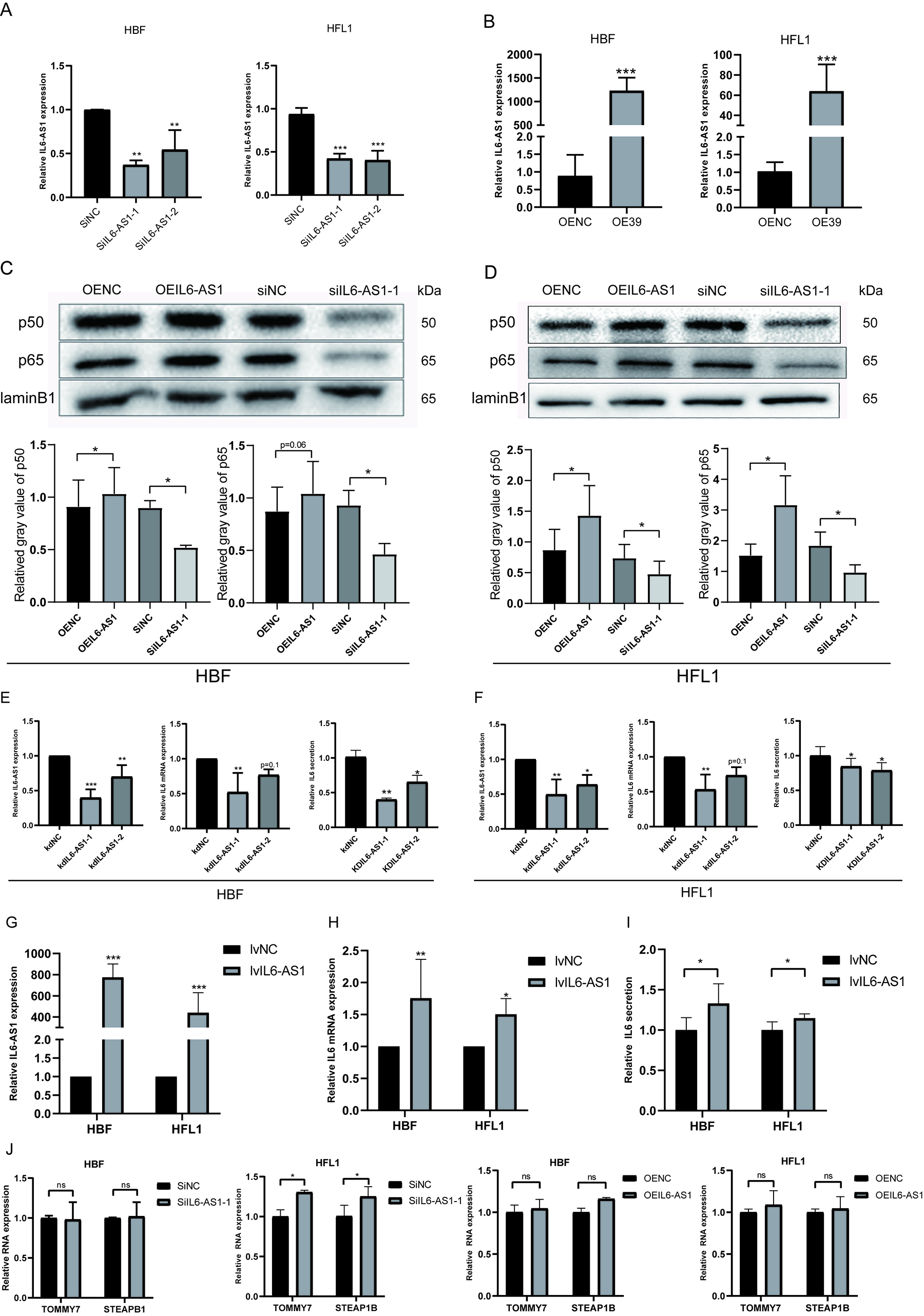

Supplement: Supplementary file 4 — Supporting Information [file CTM2-11-e479-s006.tif]

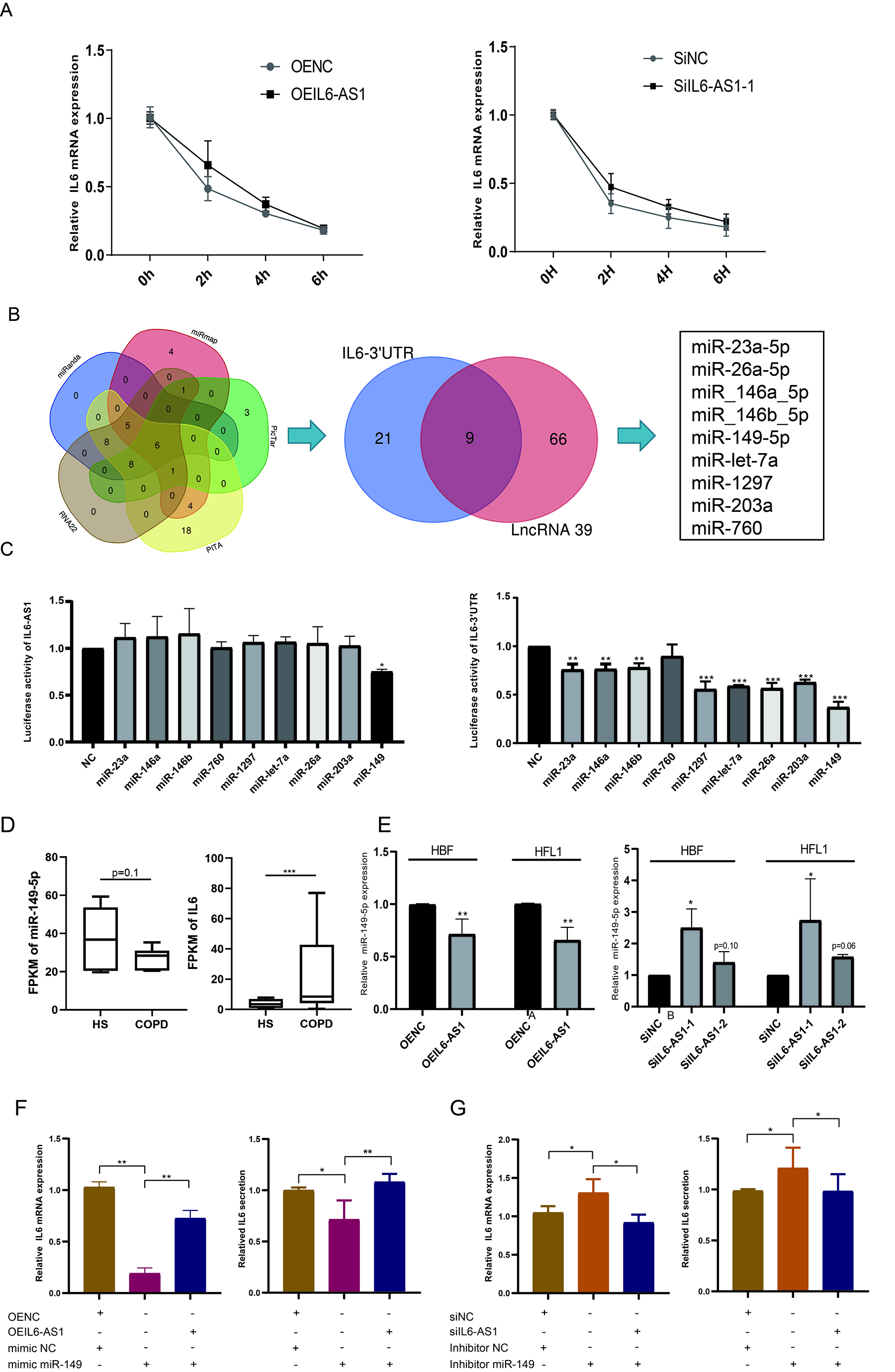

Supplement: Supplementary file 5 — Supporting Information [file CTM2-11-e479-s008.tif]

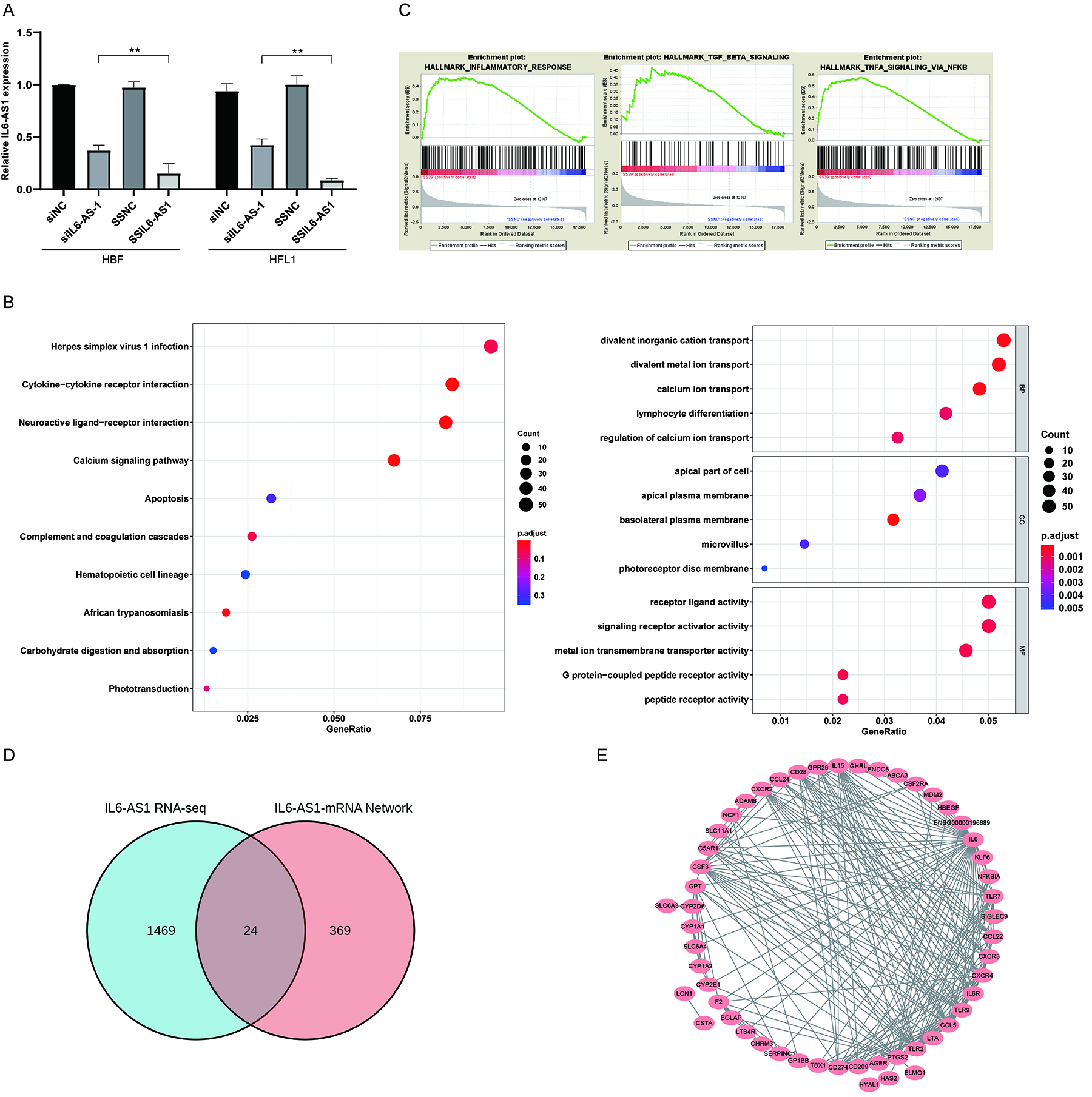

Supplement: Supplementary file 6 — Supporting Information [file CTM2-11-e479-s009.tif]

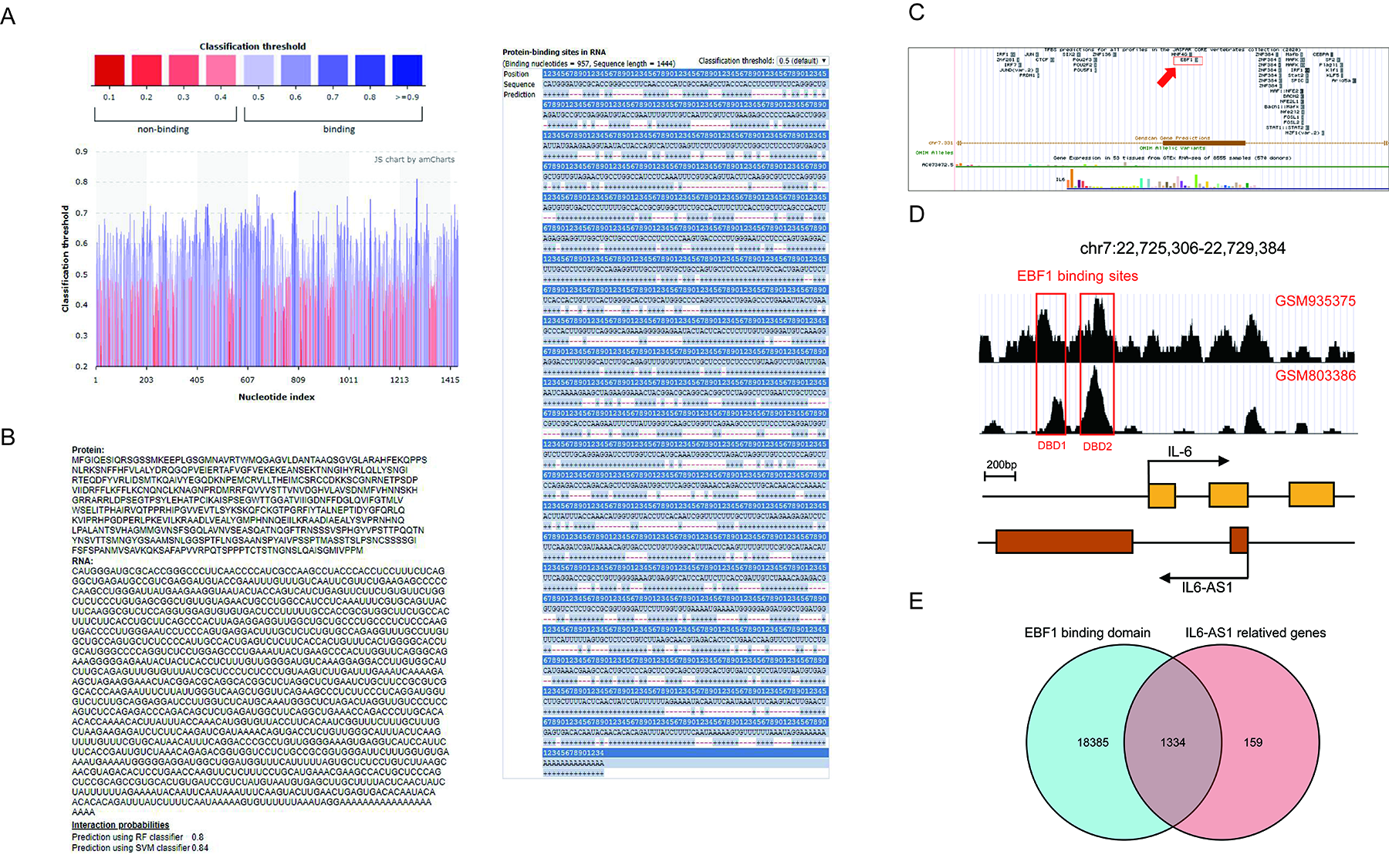

Supplement: Supplementary file 7 — Supporting Information [file CTM2-11-e479-s002.tif]

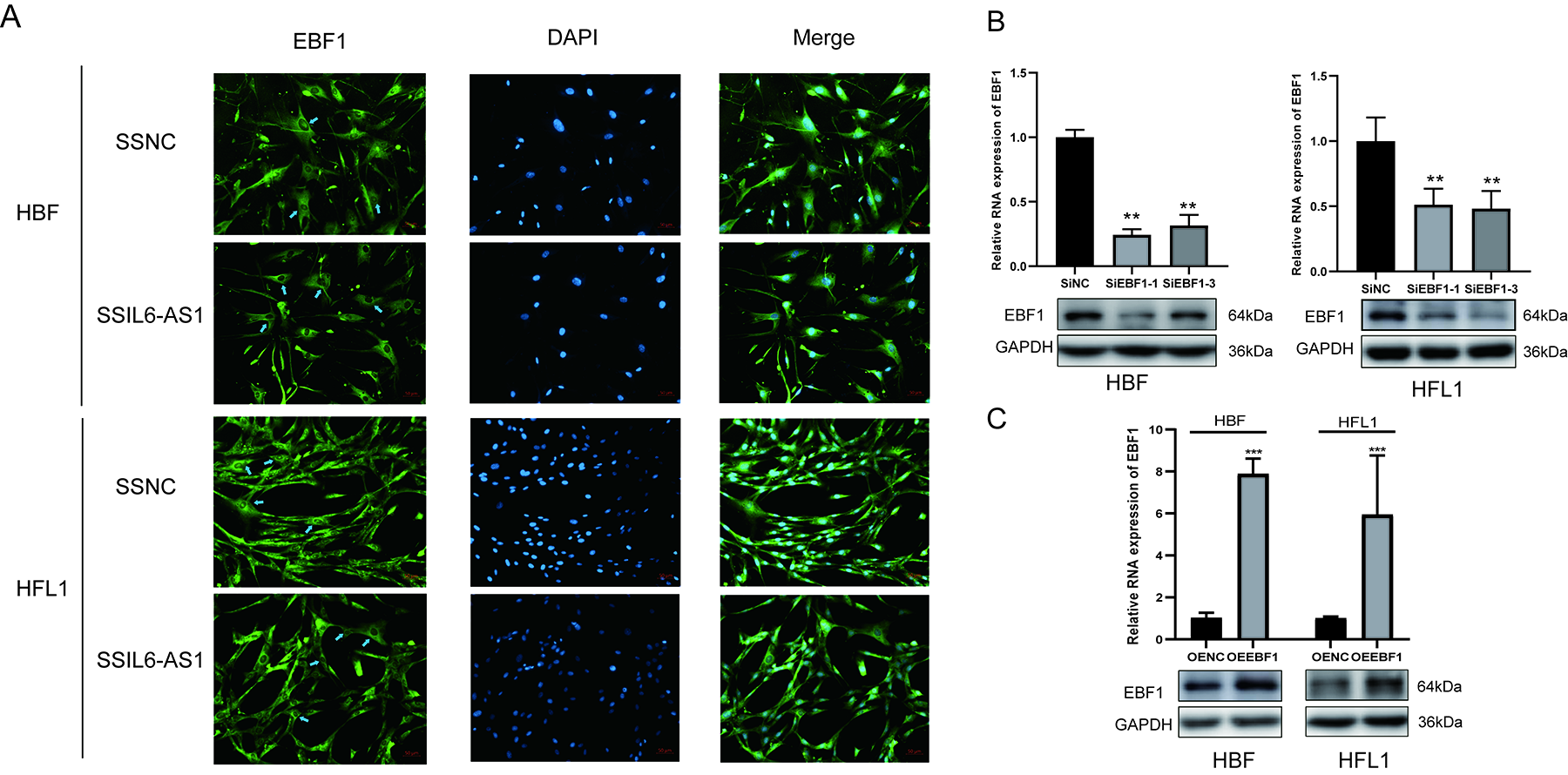

Supplement: Supplementary file 8 — Supporting Information [file CTM2-11-e479-s001.tif]

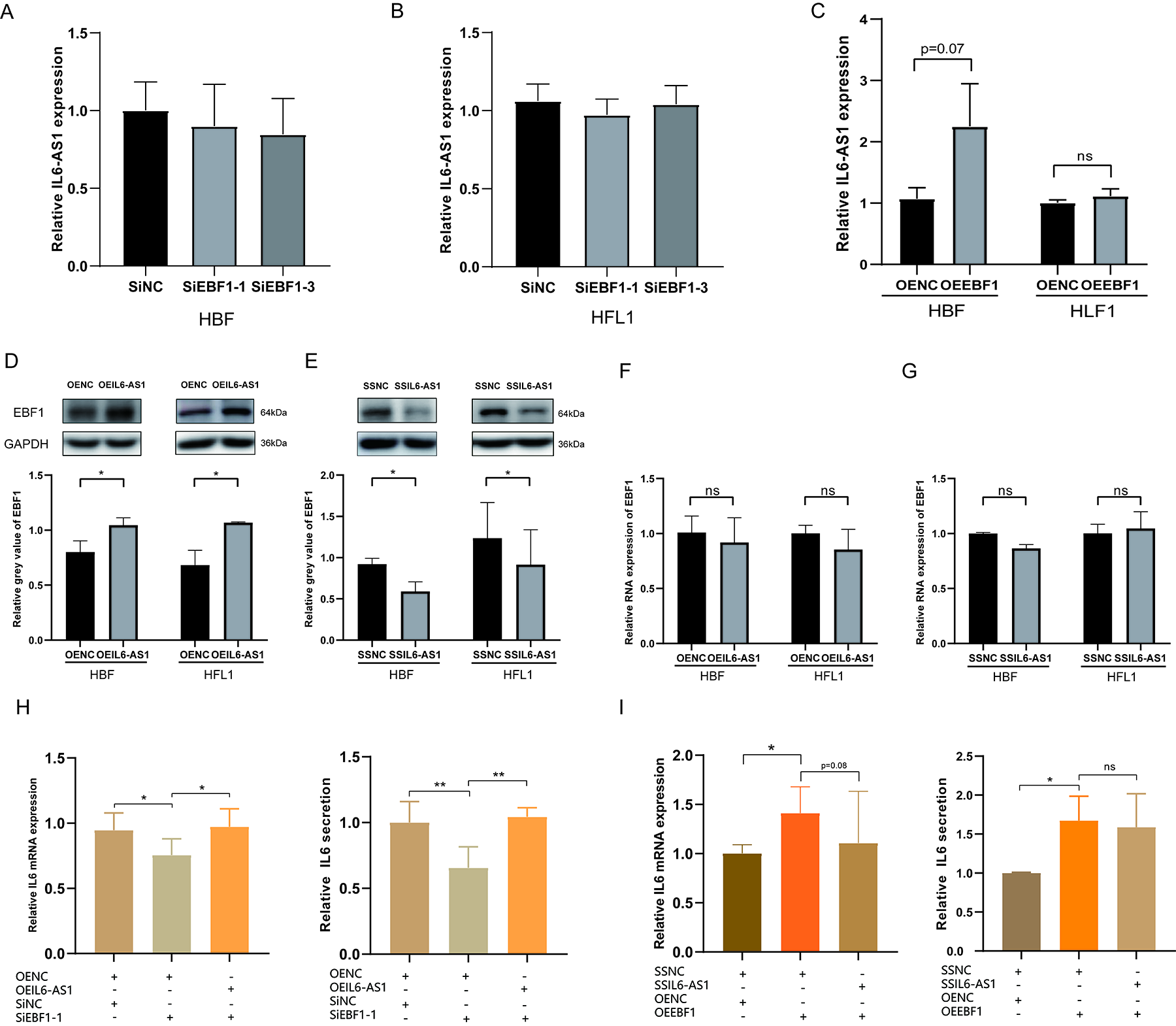

Supplement: Supplementary file 9 — Supporting Information [file CTM2-11-e479-s010.tif]

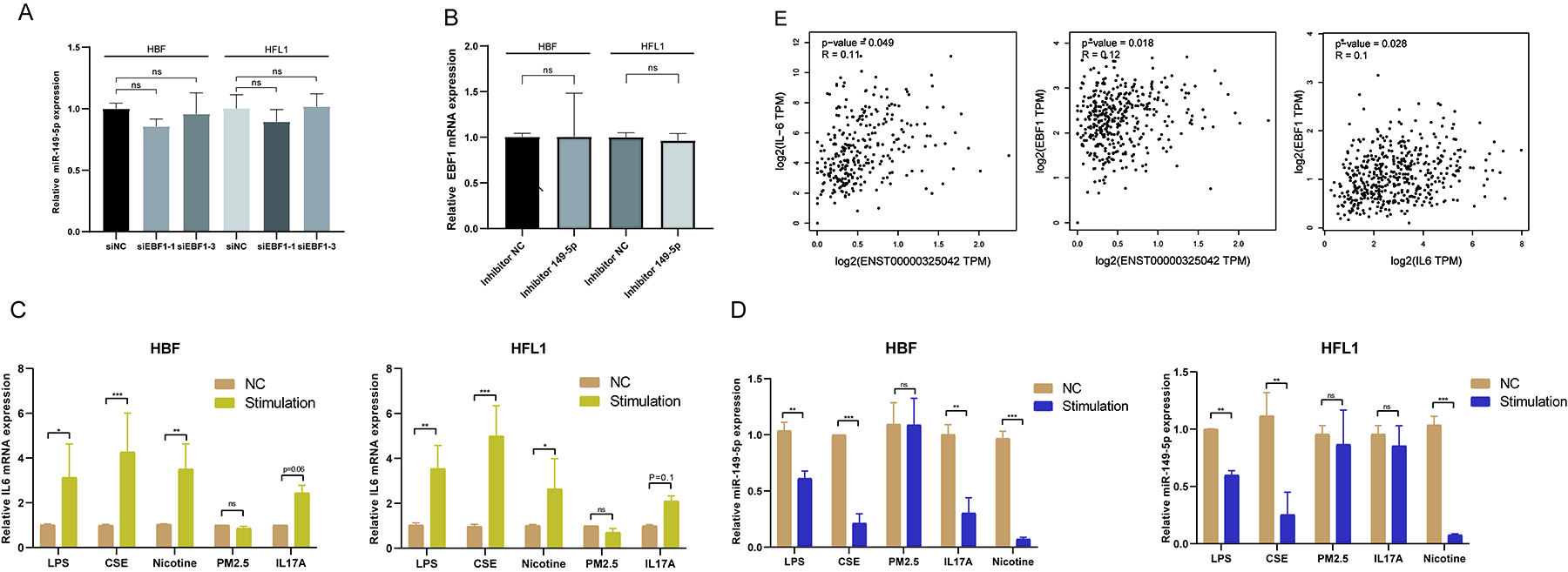

Supplement: Supplementary file 10 — Supporting Information [file CTM2-11-e479-s011.tif]
