# Supplementary material for: Long noncoding RNA IL6‐AS1 is highly expressed in chronic obstructive pulmonary disease and is associated with interleukin 6 by targeting miR‐149‐5p and early B‐cell factor 1
Source: Clin Transl Med. 2021 Jul 19;11(7):e479. doi: 10.1002/ctm2.479 (PMC8288003; doi:10.1002/ctm2.479)
Supplement: Supplementary file 11 — Supporting Information [file CTM2-11-e479-s004.docx]

Supplementary Materials


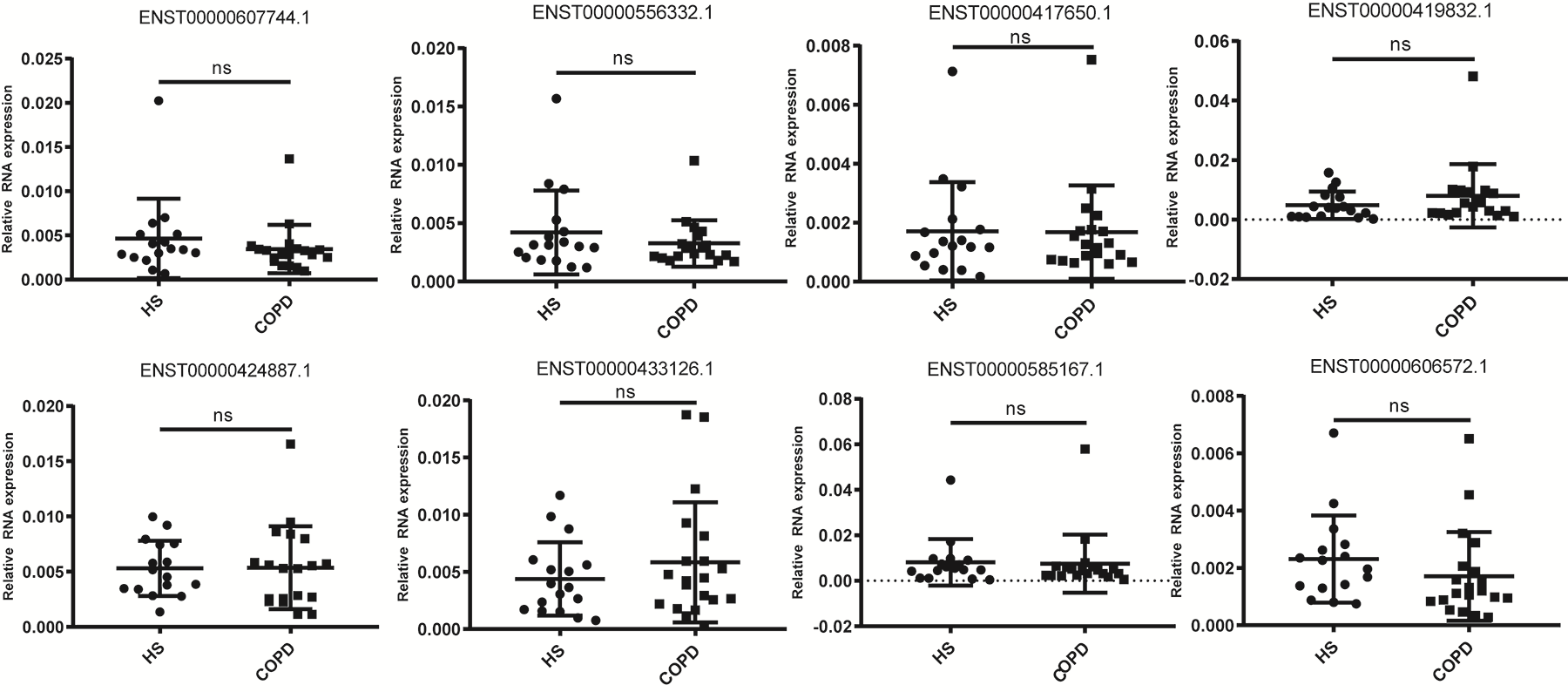


**Supplementary Fig. 1.** qRT-PCR analysis of the other eight long non-coding RNAs (lncRNAs) ENST00000417650, ENST00000424887, ENST00000433126, ENST00000556332, ENST00000585167, ENST00000606572, ENST00000607744, and ENST00000419832.1 in samples from 17 smokers with non-COPD and 19 chronic obstructive pulmonary disease patients. P values were calculated by unpaired two-tailed t test. **P* < 0.05.


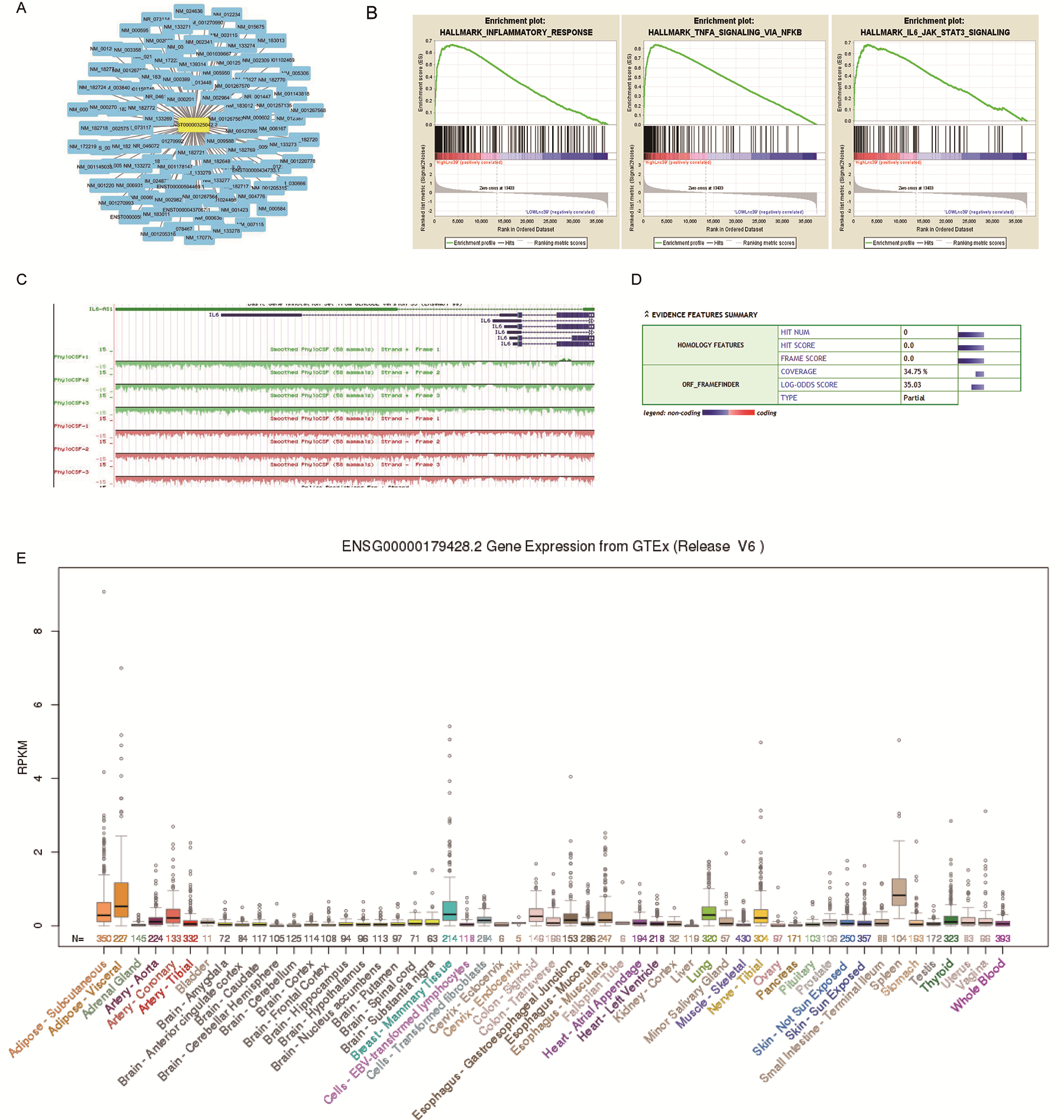


**Supplementary Fig. 2. A** IL6-AS1-related lncRNA-mRNA co-expression networks were constructed using weighted gene co-expression network analysis based on Pearson correlation coefficients. **B** Gene-set enrichment analysis of the long non-coding RNA (lncRNA)-mRNA co-expression network. **C, D** Coding potential of IL6-AS1 was predicted by PhyloCSF (https://github.com/mlin/PhyloCSF/wiki) (**C**) and the Coding Potential Calculator (http://cpc2.cbi.pku.edu.cn/) (**D**). **E** IL6-AS1 expression in various human tissues in the Genotype-Tissue Expression (GTEx) project.


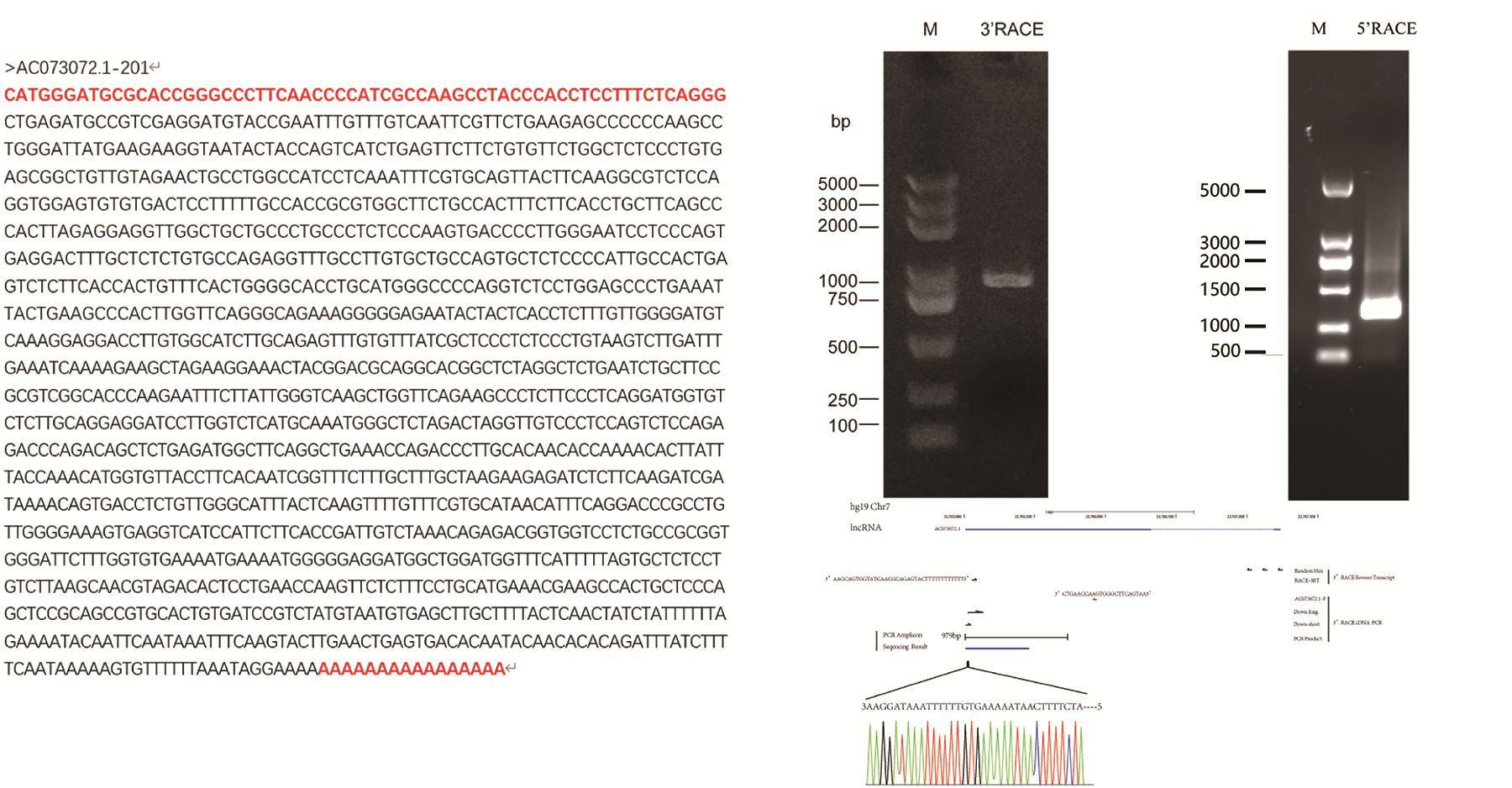


**Supplementary Fig. 3** Full-length cDNA sequence of IL6-AS1 was obtained by rapid amplification of cDNA ends (RACE). Red represents the extended sequence compared against the sequence provided in the NCBI Databank.


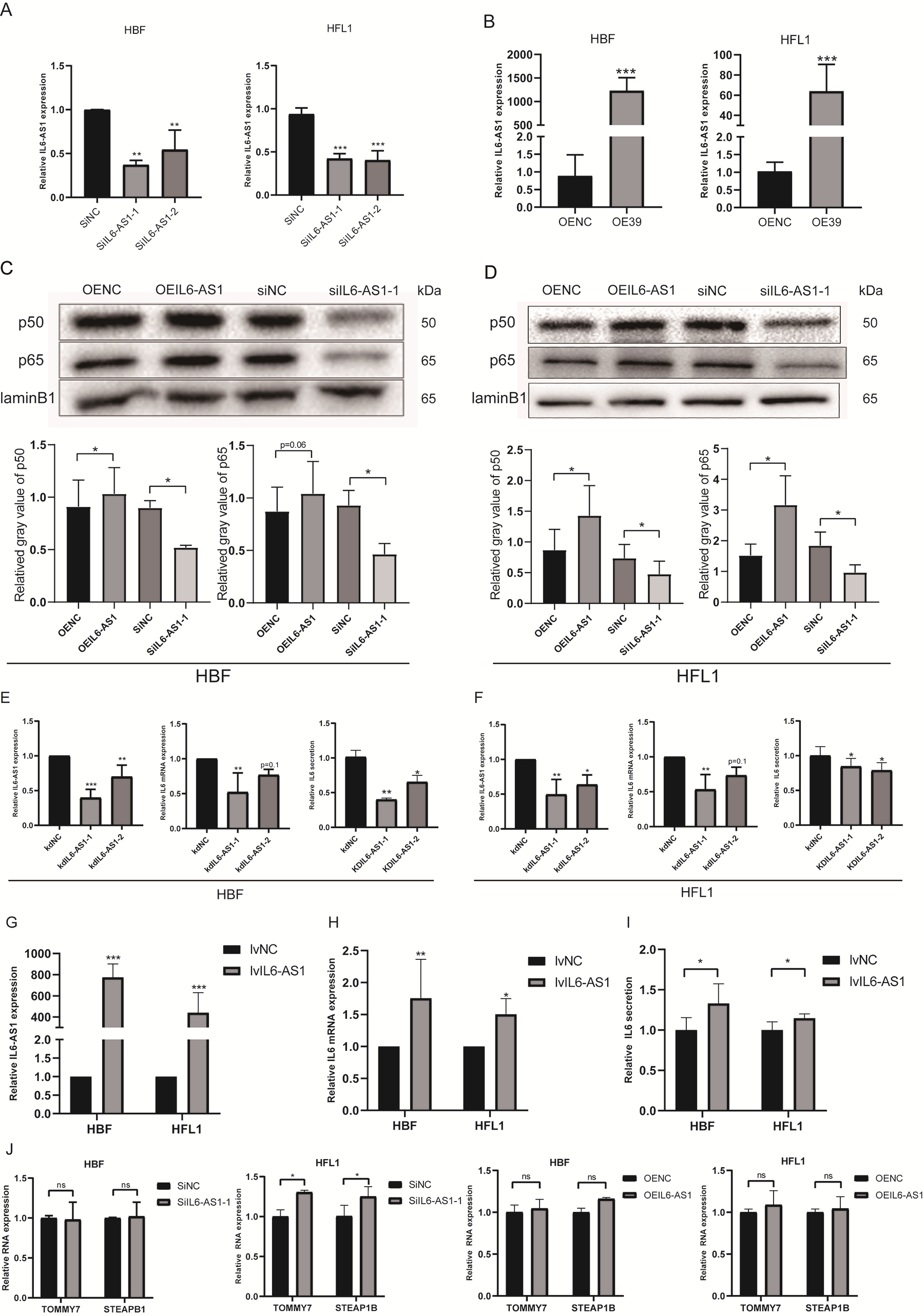


**Supplementary Fig. 4.** **A** qRT-PCR analysis of IL6-AS1 expression following knockdown of IL6-AS1 by two siRNAs in HBF and HFL1 cells. (n=5 biological replicates, one-way ANOVA).

**B** qRT-PCR analysis of IL6-AS1 expression after transfection with an overexpression vector in HBF and HFL1 cells (one-way ANOVA, n=5 biological replicates).

**C, D** Western blot analysis of p50 and p65 nuclear translocation following transfection with IL6-AS1 overexpression vector or IL6-AS1 siRNA (SiIL6-AS1-1) in HBF cells (**C**) and HFL1 cells (**D**). Lamin B1 was used as a housekeeping gene (one-way ANOVA, n=3 biological replicates).

**E-F** qRT-PCR analysis of IL6-AS1 expression following knockdown of IL6-AS1 by lentivirus-mediated RNA interference in HBF cells and HFL1 cells, qRT-PCR analysis of IL-6 expression following knockdown of IL6-AS1 by lentivirus-mediated RNA interference, ELISA analysis of IL-6 expression following knockdown of IL6-AS1 by lentivirus-mediated RNA interference as indicated (one-way ANOVA, n=3 biological replicates).

**G** qRT-PCR analysis of IL6-AS1 expression following overexpression of IL6-AS1 by lentivirus-mediated overexpression vector in HBF cells and HFL1 cells (one-way ANOVA, n=3 biological replicates).

**H, I** qRT-PCR (**H**) and ELISA (**I**) analysis of IL-6 expression following overexpression of IL6-AS1 by lentivirus in HBF and HFL1 cells (two-tailed t test, n=3 biological replicates).

**J** qRT-PCR analysis of the expression of the adjacent genes TOMM7 and STEAP1B following transfection with IL6-AS1 siRNA (SiIL6-AS1-1) or an IL6-AS1 overexpression vector in HBF and HFL1 cells (n=3 biological replicates, two-tailed t test).

Data information: Error bars represent means ± SD, **P < 0.05, **P < 0.01 and ***P < 0.001.


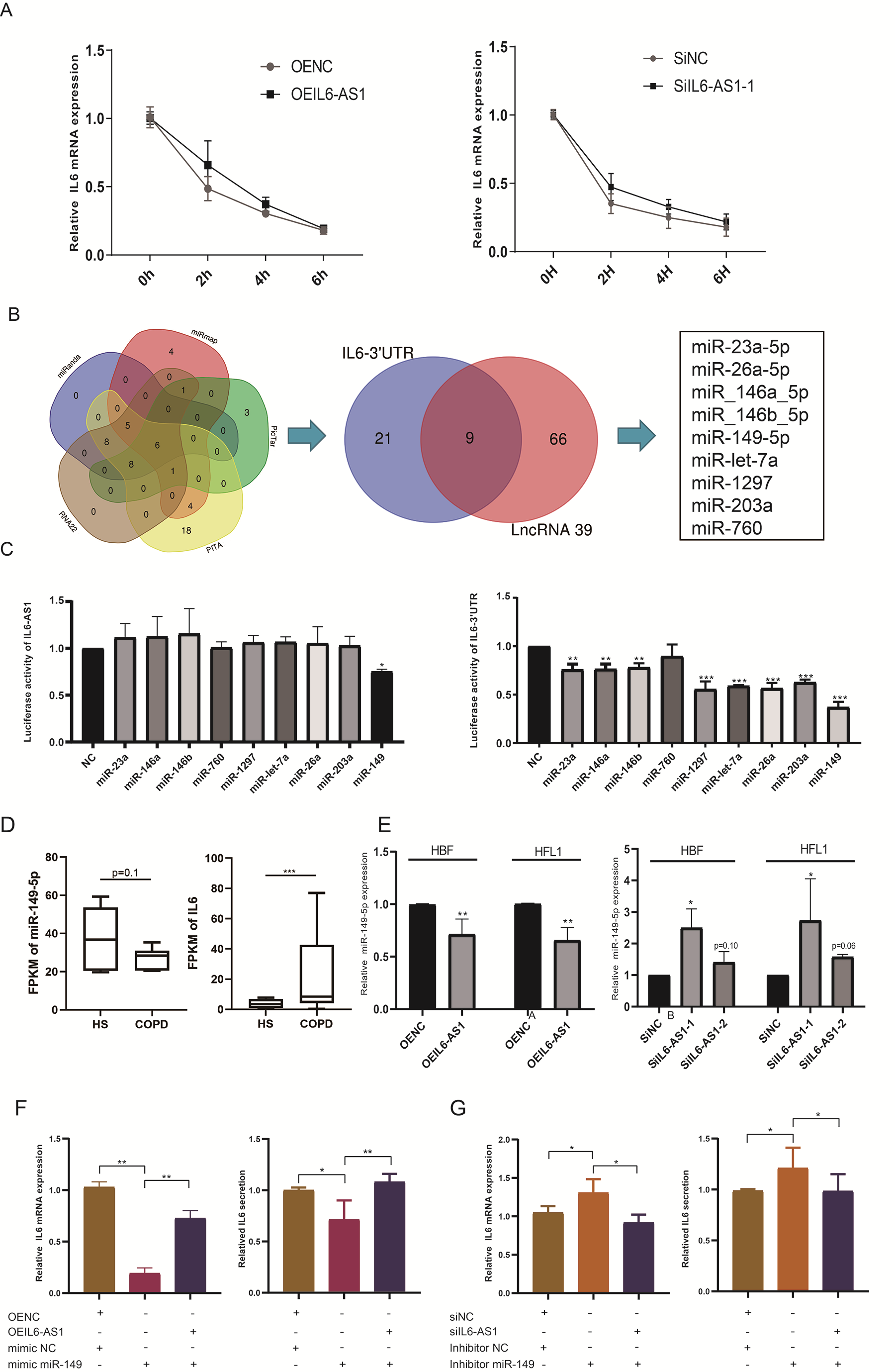


**Supplementary Fig. 5. A** HBF cells were treated with actinomycin D (Act D, 1 µg/mL) for the indicated time following transfection with IL6-AS1 overexpression vector or SiIL6-AS1-1. Interleukin (IL) 6 expression was measured by qRT-PCR. (one-way ANOVA, n=3 biological replicates.)

**B** Bioinformatics analysis of the predicted target miRNA by interleukin (IL) 6 and IL6-AS1 using five miRNA prediction databases (RNA22, PITA, PicTar, miRmap, and miRanda). The intersection of predicted miRNAs for both IL-6 and IL6-AS1 are shown.

**C** Wild-type IL6-AS1 sequences and IL6-3′UTR sequences were cloned into pGL4 vectors and co-transfected with mimics of selected miRNAs (miR-23a-5p, miR-26-5p, miR-146a-5p, miR-146b-5p, miR-let-7a, miR-1297, miR-203a, miR-760, and miR-149-5p) or miR-NC into 293T cells. The relative luciferase activity was normalized against *Renilla* luciferase activity. (one-way ANOVA, n=3 biological replicates)

**D** Fragments per kilobase of transcript per million mapped reads for interleukin (IL) 6 nd miR-149-5p in samples from chronic obstructive pulmonary disease patients.

**E** qRT-PCR analysis of miR-149-5p expression following overexpression or knockdown of IL6-AS1 in HBF and HFL1 cells (one-way ANOVA, n=3 biological replicates).

**F** IL-6 expression was measured in HFL1 cells co-transfected with OEIL6-AS1 and miR-149-5p mimic by qRT-PCR and ELISA (one-way ANOVA, n=5 biological replicates).

**G** IL-6 expression was measured in HFL1 cells co-transfected with an siIL6-AS-1 and miR-149-5p inhibitor by qRT-PCR and ELISA (one-way ANOVA, n=5 biological replicates).

Data information: Error bars represent means ± SD. **P* < 0.05, ***P* < 0.01 and ****P* < 0.001.


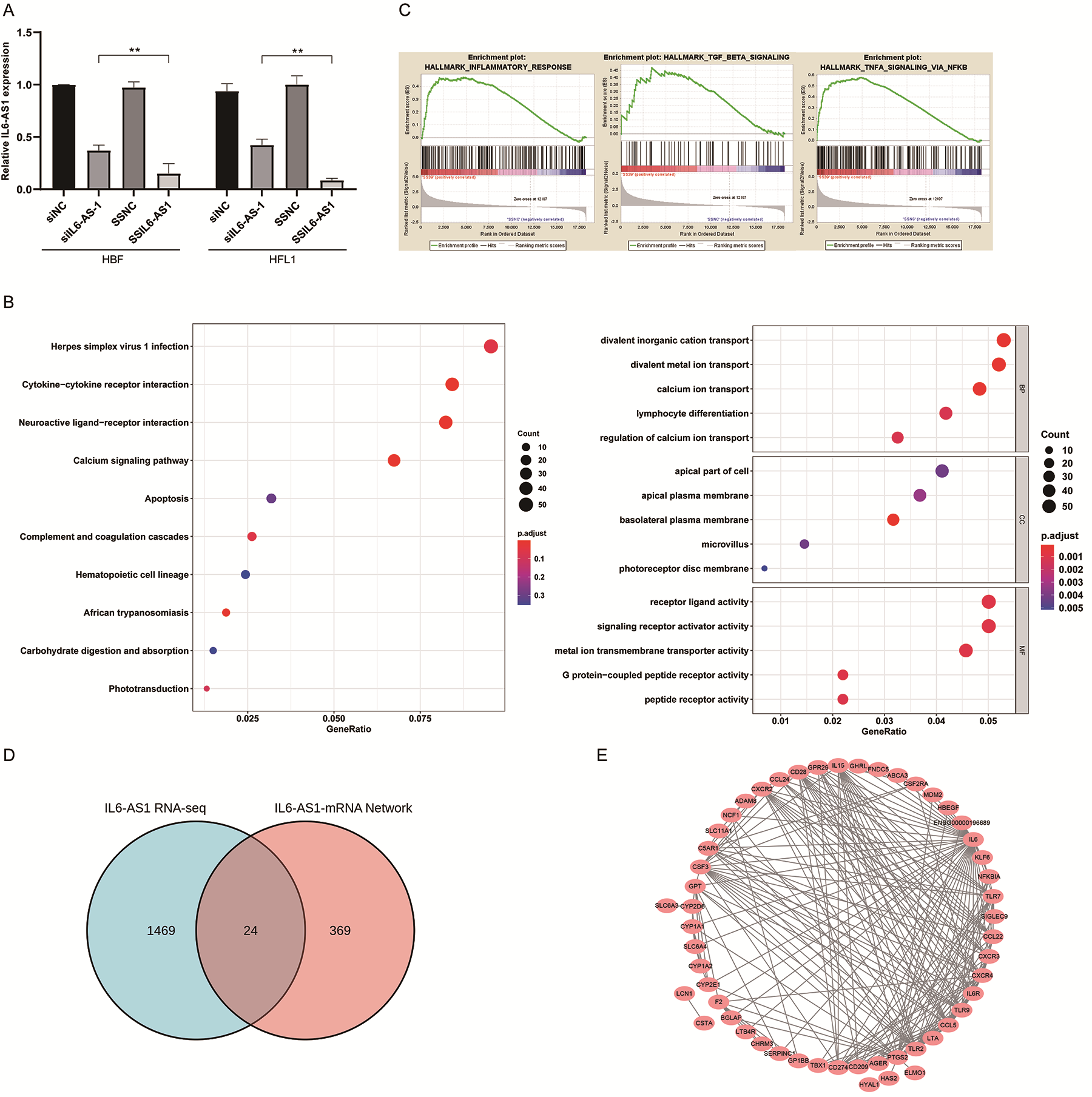


**Supplementary Fig. 6. A** qRT-PCR analysis of IL6-AS1 expression following knockdown of IL6-AS1 by siRNA (siIL6-AS1) or Smart Silencer (SSIL6-AS1) in HBF and HFL1 cells. (Error bars represent means ± SD, n=5 biological replicates, two-way ANOVA, ***P* < 0.01)

**B** KEGG and GO analysis of differentially expressed genes identified by polyA mRNA sequencing after IL6-AS1 knockdown by SSIL6-AS1.

**C** GSEA analysis of differentially expressed genes identified by polyA mRNA sequencing after IL6-AS1 knockdown by SSIL6-AS1.

**D** Venn diagram showing the overlapping genes between the IL6-AS1 RNA-seq and IL6-AS1-mRNA co-expression network DEGs.

**E** Schematic representation of the PPI network of genes that intersect between IL6-AS1 RNA-seq DEGs and COPD-related genes.


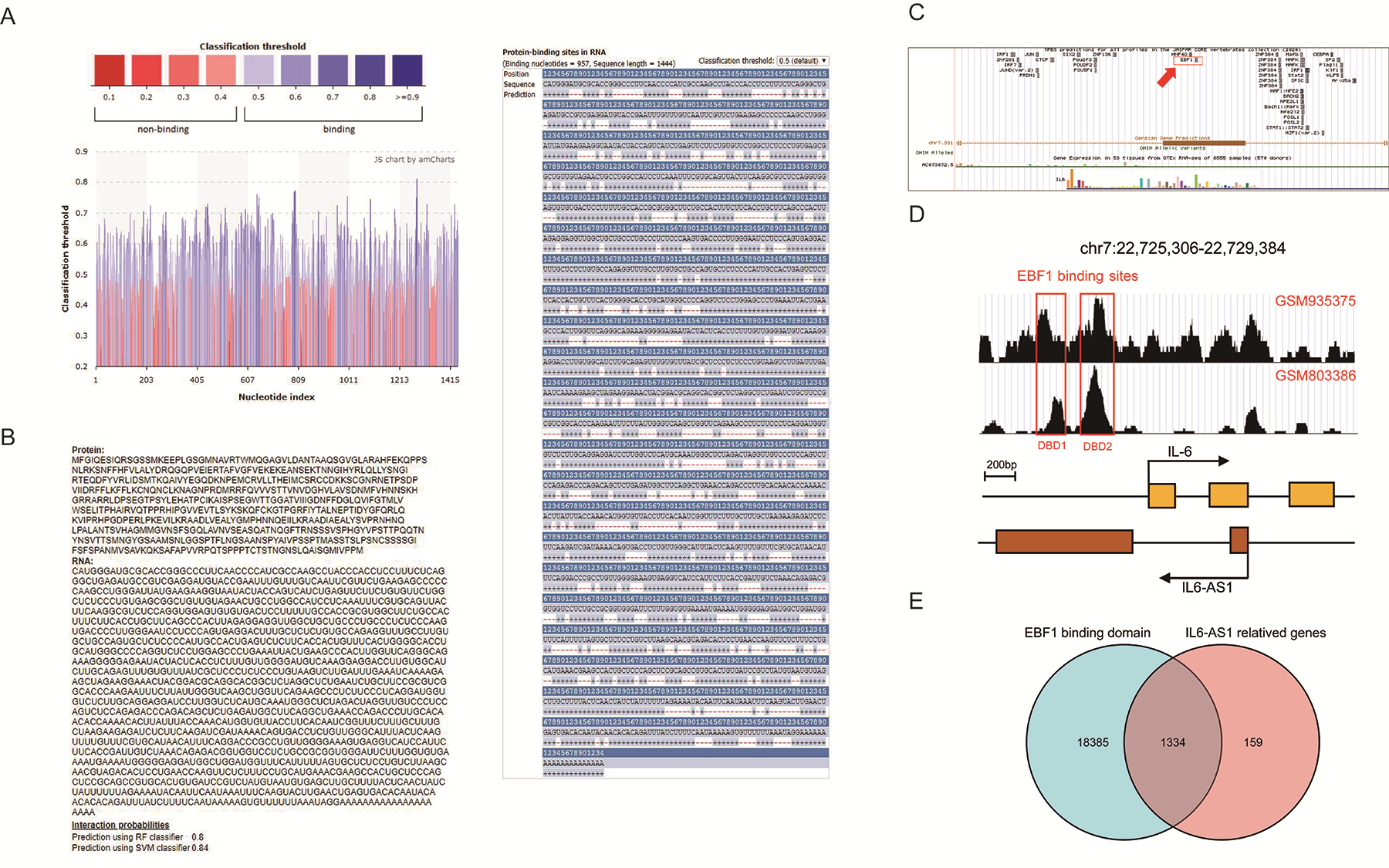


**Supplementary Fig. 7. A, B** The binding of lncRNA to EBF1 was predicted by RNAInter (**A**) (http://www.rna-society.org/raid/PRIdictor.html) and RPISeq (**B**) (http://pridb.gdcb.iastate.edu/RPISeq/index.html).

**C** Transcription factor binding site prediction on the interleukin (IL) 6 promoter by JASPAR (http://jaspar.genereg.net/). Red arrow indicates the EBF1 transcription factor.

**D** Schematic showing the ChIP-seq data (GSM803386 and GSM 935375) for EBF1 binding upstream of IL-6.

**E** The intersection of potential EBF1-targeted genes and IL6-AS1-related genes.


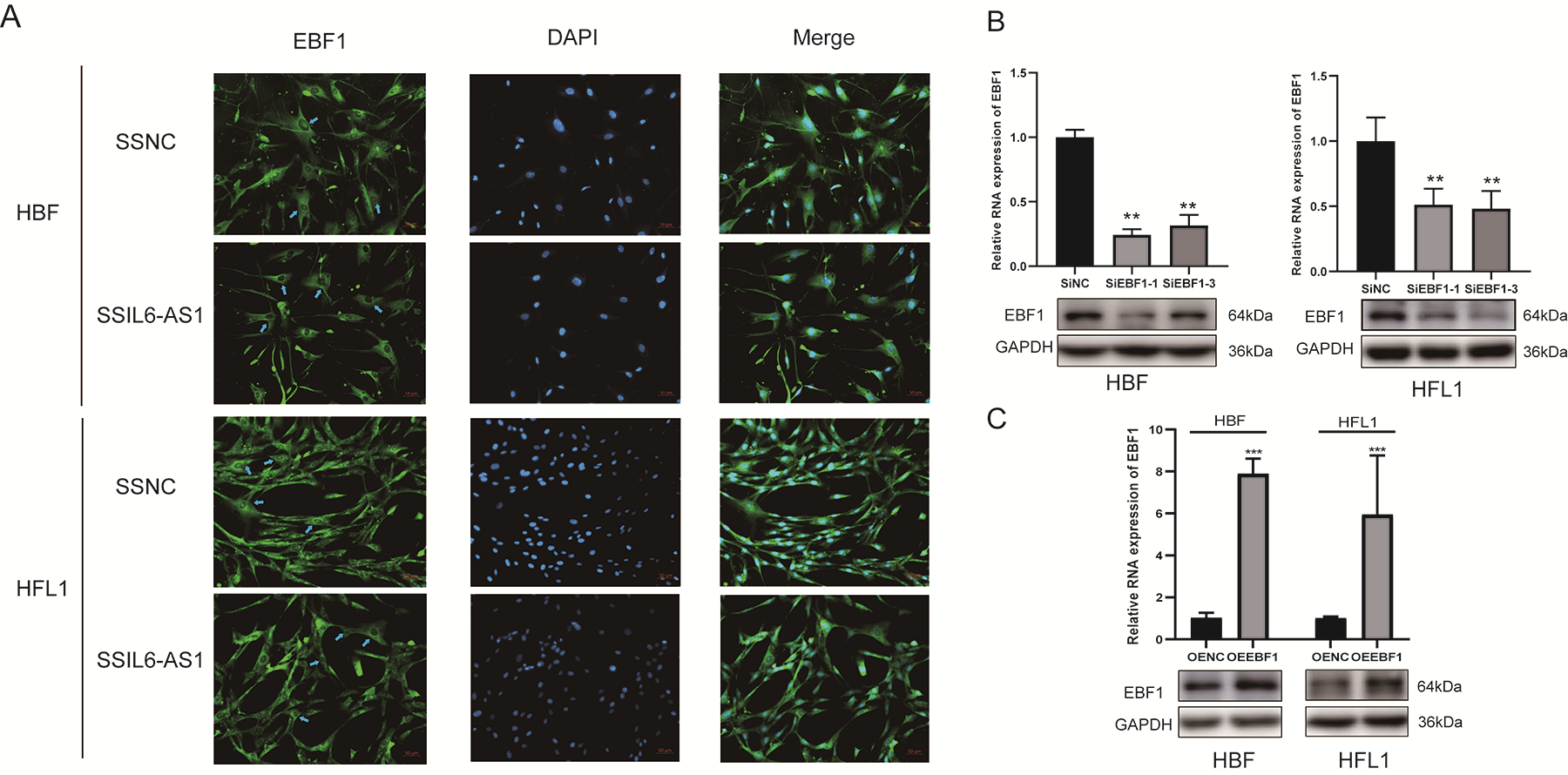


**Supplementary Fig. 8. A** Immunofluorescence analysis of EBF1 nuclear translocation following transfection with a IL6-AS1 silencer (SSIL6-AS1) in HBF and HFL1 cells. Green fluorescence represents the EBF1 proteins and blue fluorescence (DAPI) indicates nuclear DNA.

**B** qRT-PCR and western blot analysis of EBF1 expression following transfection with two siRNAs (siEBF1-1 and siEBF1-3) in HBF cells and HFL1 cells (one-way ANOVA, n=3 biological replicates).

**C** qRT-PCR and western blot analysis of EBF1 expression following transfection with an EBF1 overexpression vector in HBF and HFL1 cells (one-way ANOVA, n=3 biological replicates).

Data information: Error bars represent means ± SD. ***P* < 0.01.


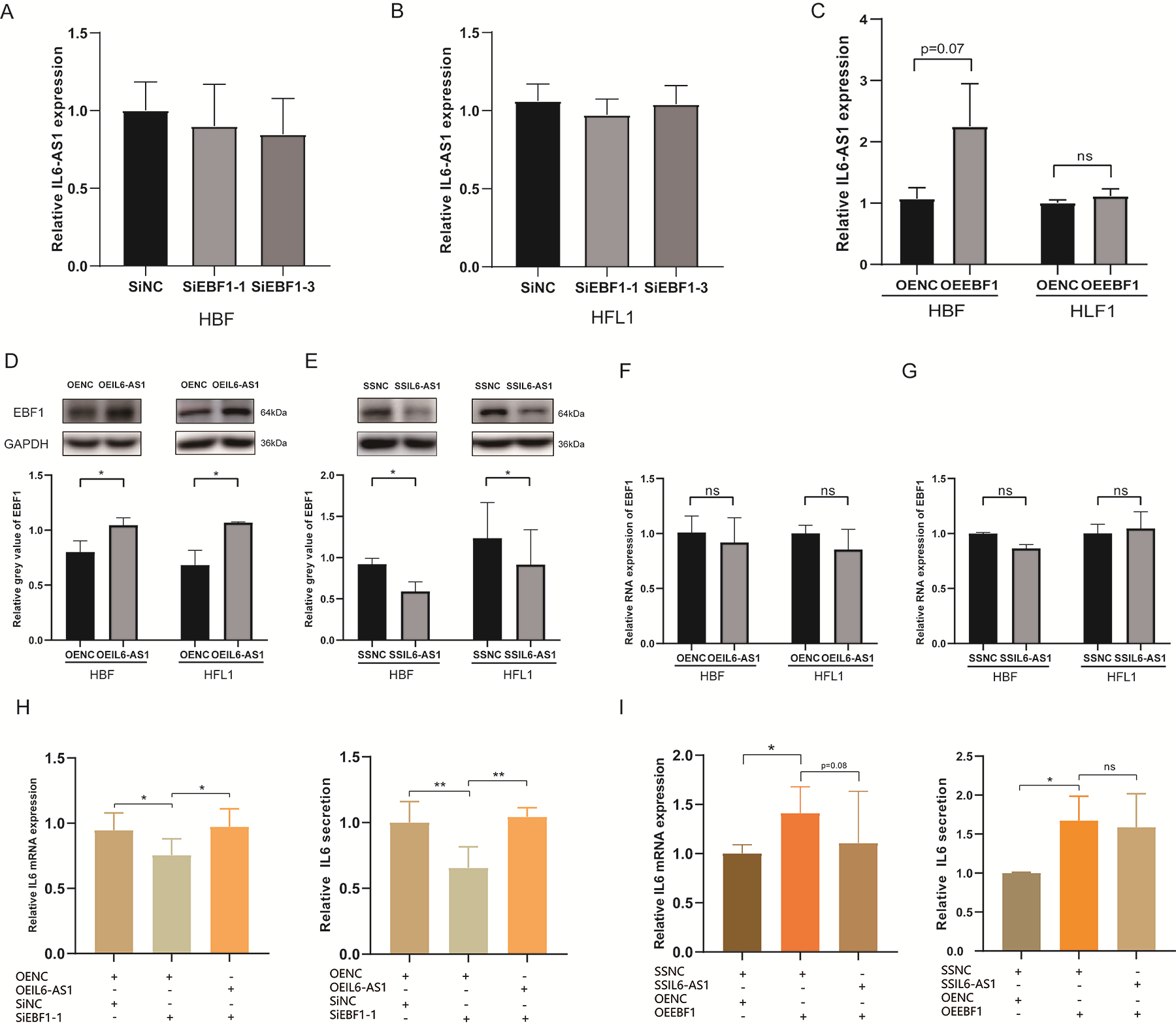


**Supplementary Fig. 9. A**, **B** qRT-PCR analysis of IL6-AS1 expression following transfection with EBF1 siRNA in HBF cells (**A**) and HFL1 cells (**B**) (one-way ANOVA, n=3 biological replicates).

**C** qRT-PCR anaylsis of IL6-AS1 expression following transfection with an EBF1 overexpression vector in HBF and HFL1 cells (one-way ANOVA, n=3 biological replicates).

**D**, **E** Western blot analysis of EBF1 expression following transfection with an IL6-AS1 overexpression vector (**D**) orIL6-AS1 silencer (SSIL6-AS1) (**E**) in HBF and HFL1 cells. (paired two-tailed t test, n=3 biological replicates).

**F**, **G** qRT-PCR analysis of EBF1 expression following transfection with an or IL6-AS1 overexpression vector (**F**) and IL6-AS1 silencer (SSIL6-AS1) (**G**) in HBF and HFL1 cells. (one-way ANOVA, n=3 biological replicates).

**H** Expression of IL-6 in HBF cells following co-transfection with IL6-AS1 overexpression vector and EBF1 siRNA (SiEBF1-1), determined by qRT-PCR and ELISA (one-way ANOVA, n=4 biological replicates).

**I** Expression of IL-6 in HBF cells following co-transfection with an IL6-AS1 Smart Silencer (SSIL6-AS1) and EBF1 overexpression vector, determined by qRT-PCR and ELISA (one-way ANOVA, n=4 biological replicates).

Data information: Error bars represent means ± SD. **P* < 0.05 and ***P* < 0.01.


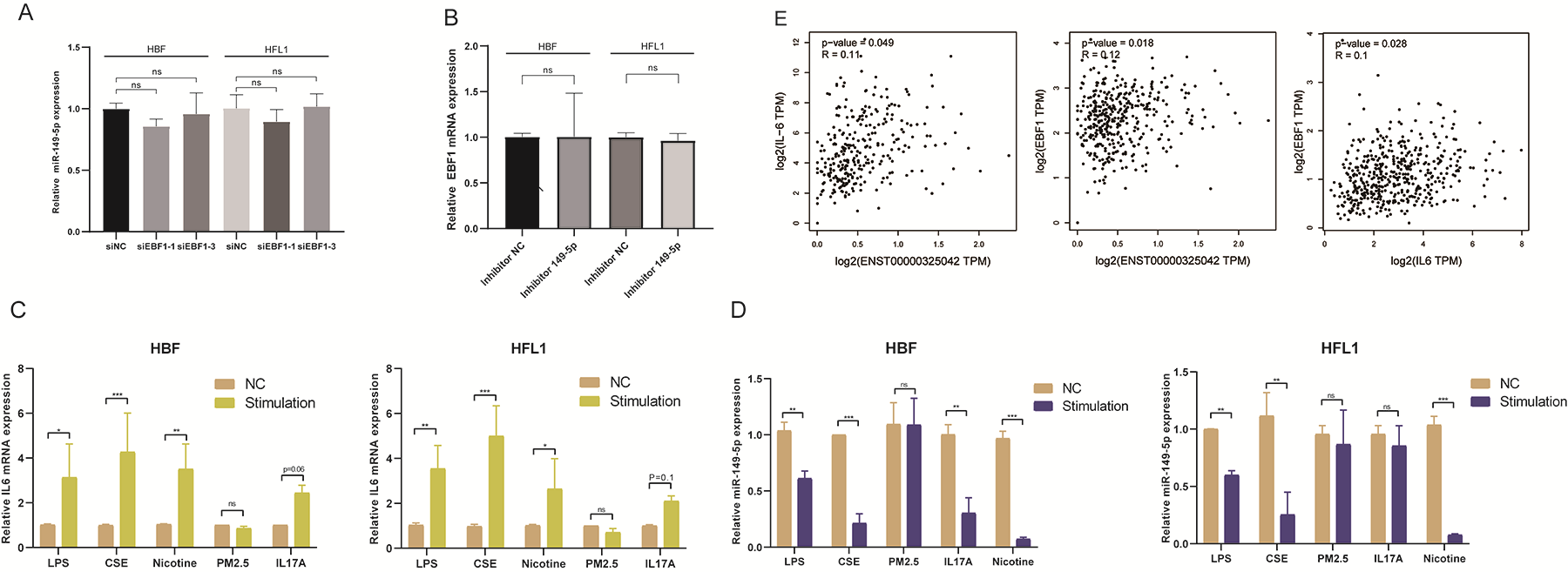


**Supplementary Fig. 10. A** qRT-PCR analysis of miR-149-5p expression after transfection of HBF and HFL1 cells with two EBF1 siRNAs (siEBF1-1 or siEBF1-3) (one-way ANOVA, n=3 biological replicates).

**B** qRT-PCR analysis of EBF1 expression after transfection of HBF and HFL1 cells with miR-149-5p inhibitor (one-way ANOVA, n=3 biological replicates).

**C, D** HBF and HFL1 cells were separately exposed to LPS (500 ng/ml), cigarette smoke extract (CSE, 0.015%), PM_2.5_ (2 µg/ml), IL17A (200 ng/ml) and nicotine (10 μM) for 24 hours. qRT-PCR analysis of IL-6 **(C)** and miR-149-5p **(D)** expression (two-way ANOVA, n=3 biological replicates).

**E** Correlation analysis of gene expression in normal lung tissues between IL6-AS1/IL-6, IL6-AS1/EBF1, and IL6/EBF1 by Gene Expression Profiling Interactive Analysis (GEPIA2, http://gepia2.cancer-pku.cn).

**Supplementary Table 1.** **Clinical characteristics of the subjects for sample validation**

|  | **Smokers without COPD** | **COPD** |
| --- | --- | --- |
| **N** | 16 | 19 |
| **Sex, M/F^*^** | 14/2 | 14/5 |
| **Age, year^*^** | 55.56±9.19 | 63.28±11.07 |
| **BMI, kg/m^2*^** | 23.42±2.87 | 23.70±3.42 |
| **FEV1, %pred^#^** | 100.89±18.34 | 84.28±22.09 |
| **FEV1/FVC%^#^** | 81±7.83 | 64.94±5.92 |

*P>0.05, #P<0.05. Data are presented as the means ± SDs.

**Supplementary Table2. The genes in the intersection of the DEGs in IL6-AS1 RNA-seq and the genes in lncRNA-mRNA co-expression network.**

| **GeneSymbol** |  |  |  |  |
| --- | --- | --- | --- | --- |
| LTA | ZNF460 | CSF3 | NFKBIA | HAS2 |
| EID3 | TNFAIP3 | BIRC3 | RHOB | PTGS2 |
| FAM150B | IL1RL2 | CLCF1 | CSRNP1 | SGPP2 |
| LIF | TNIP3 | IRAK2 | ZNF490 | DCUN1D3 |
| MGAM | IL6 | FCAR | PPP1R3B |  |

**Supplementary Table3.** **The genes in the intersection of the DEGs in IL6-AS1 RNA-seq and the genes related to COPD.**

| **GeneSymbol** | |  |  |  |  |
| --- | --- | --- | --- | --- | --- |
| IL6 | CYP2E1 | CSF3 | PTGS2 | CYP1A2 | CD209 |
| CCL5 | TRPV1 | BGLAP | LTB4R | GHRL | CCL24 |
| CXCR3 | ABCA3 | TLR9 | IL6R | NFKBIA | HAS2 |
| TLR2 | LTA | C5AR1 | CD28 | ADAM8 | ESM1 |
| CHRM3 | NPPA | CXCR4 | HBEGF | SLC6A3 | CCL22 |
| CXCR2 | F2 | IL15 | PDE4C | SIGLEC9 | CDH23 |
| CYP1A1 | GPT | AGER | NCF1 | KLF6 | ELMO1 |
| SLC6A4 | SLC4A1 | CD274 | TBX1 | FNDC5 | ASTN2 |
| CYP2D6 | CSF2RA | SLC11A1 | GP1BB | NME9 | NR4A3 |
| CCR6 | SLC52A3 | SERPINC1 | LCN1 | MDM2 | HYAL1 |
| CSTA | TLR7 |  |  |  |  |

**Supplementary Table 4. ChIP-seq results of EBF1 binding sites on IL-6 chormatin from ENCODE database**

**GSM935375**

| **chrom** | **txStart** | **txEnd** | **strand** |
| --- | --- | --- | --- |
| chr7 | 22725888 | 22732001 | + |
| chr7 | 22727162 | 22731020 | + |
| chr7 | 22727199 | 22731997 | + |
| chr7 | 22727199 | 22731997 | + |
| chr7 | 22727199 | 22731997 | + |

**GSM803386**

| **chrom** | **txStart** | **txEnd** | **strand** |
| --- | --- | --- | --- |
| chr7 | 22727199 | 22731997 | + |
| chr7 | 22727199 | 22731997 | + |
| chr7 | 22727199 | 22731997 | + |
| chr7 | 22727162 | 22731020 | + |
| chr7 | 22725888 | 22732001 | + |

**Supplementary Table5.** **Primers used in experiments**

| **Primer name** | **Forward primer (5’-3’)** | **Reverse primer (5’-3’)** | **Application** |
| --- | --- | --- | --- |
| IL6-AS1  GAPDH  IL6 | GCATAACATTTCAGGACCCGC  CAGCCTCAAGATCATCAGCA  AAGCCAGAGCTGTGCAGATGAGTA | GGAGCAGTGGCTTCGTTTCA  ACAGTCTTCTGGGTGGCAGT  TGTCCTGCAGCCACTGGTTC | qRT-PCR  qRT-PCR  qRT-PCR |
| IL8 | AACTGAGAGTGATTGAGAGTGG | ATGAATTCTCAGCCCTCTTCAA | qRT-PCR |
| NFKB1  CCL2 | GCCTCCACAAGGCAGCAAATA  CTTCTGTGCCTGCTGCTCATA | CACCACTGGTCAGAGACTCGGTAA  CTTTGGGACACTTGCTGCTG | qRT-PCR  qRT-PCR |
| CCR7  TOMM7  STEAP1B  U6  EBF1  NEAT1  RELA | AGCACCTGTGAGCTCAGTAAGCAA  GGTTGCTGTAAGGGGTCCTC  TGTCTTACGCAATGAGGCGA  CTCGCTTCGGCAGCACATATA  AGCCAACAGCGAAAAGACCA  CCAGTTTTCCGAGAACCAAA  CTTCCAAGAAGAGCAGCGTG | GCCGATGAAGGCGTACAAGAA  TTCCATGCTGTCCGCTGATT  CACAGCCAACAGAGCCAGTA  ACGCTTCACGAATTTGCGTGTC  TTCGGTTGCCACAGCTTTTC  ATGCTGATCTGCTGCGTATG  GCCTGGTCCCGTGAAATACA | qRT-PCR  qRT-PCR  qRT-PCR  qRT-PCR  qRT-PCR  qRT-PCR  qRT-PCR |
| U1 | TCAAGAAGGATGCACCCCCA | ATAATACGCCCGAGTTCCCC | qRT-PCR |
| IKKβ | AACAACGGGCCGTGAAATTG | TTTGATGGGGGATGAAGGGC | qRT-PCR |
| miR-149-5p | AACAAGTCTGGCTCCGTGT | GTCGTATCCAGTGCAGGGT | qRT-PCR |
| miR-149-5p(RT) | GTCGTATCCAGTGCAGGGTCCGAGGTATTCGCACTGGATACGACGGGAGT | | RT |
| IL6pro-P1 | TATTGAAAAGATAAATCTGTGTG | AGGATGGCTGGATGGTT | ChIP-qPCR |
| IL6pro-P2 | GGCAGAGGACCACCGTCT | CTTCAGGCTGAAACCAGACC | ChIP-qPCR |
| IL6pro-P3 | TGGAGACTGGAGGGACAA | AGAGTTTGTGTTTATCGCTC | ChIP-qPCR |
| IL6pro-P4 | TTGACATCCCCAACAAAGA | CTCTCCCAAGTGACCCC | ChIP-qPCR |
| IL6pro-P5 | CGGTGGCAAAAAGGAGTCAC | GCTAGAATTTAGCGTTCCAGTTAAT | ChIP-qPCR |
| IL6pro-P6 | TCAAAAAACATAGCTTTAGCTTATT | CTGATTGGAAACCTTATTAAGATTG | ChIP-qPCR |
| IL6pro-TSS1 | TCCTTAGCCCTGGAACTGCC | GGGTGAGCTGACAGCACAGC | ChIP-qPCR |
| siIL6-AS1-1 | CAUCCUCAAAUUUCGUGCAGUUACU | AGUAACUGCACGAAAUUUGAGGAUG | siRNA |
| siIL6-AS1-2 | GAGAUGGCUUCAGGCUGAAACCAGA | UCUGGUUUCAGCCUGAAGCCAUCUC | siRNA |
| shIL6-AS1-1 | GGATGTACCGAATTTGTTTGT | ACAAACAAATTCGGTACATCC | shRNA |
| shIL6-AS1-2 | GCGTGGCTTCTGCCACTTTCT | AGAAAGTGGCAGAAGCCACGC | shRNA |
| siEBF1-1 | CCACGAGCATGAACGGATA | TATCCGTTCATGCTCGTGG | siRNA |
| siEBF1-3 | GCATGATTGTTCCTCCTAT | ATAGGAGGAACAATCATGC | siRNA |
| IL6-AS1 Smart Silencer | CTCTTCACCACTGTTTCACT | AGTGAAACAGTGGTGAAGAG | siRNA |
|  | TAAGCAACGTAGACACTCCT | AGGAGTGTCTACGTTGCTTA | siRNA |
|  | TCTATGTAATGTGAGCTTGC | GCAAGCTCACATTACATAGA | siRNA |
|  | TCTCTTCACCACTGTTTCA | TGAAACAGTGGTGAAGAGA | ASO |
|  | TGGGCATTTACTCAAGTTT | AAACTTGAGTAAATGCCCA | ASO |
|  | CATCCTCAAATTTCGTGCAGTTACT | CATCCTCAAATTTCGTGCAGTTACT | ASO |

**Supplementary Table 6. RACE primers**

| **Primer name** | **Forward primer (5’-3’)** | **Reverse primer (5’-3’)** |
| --- | --- | --- |
| 5’RACE-1(Outer) | Universal primer in SMARTer kit | AGGCGTCTCCAGGTGGAGTGTGTGACTC |
| 5’RACE-2(Inner) | Universal primer in SMARTer kit | CAGCCCACTTAGAGGAGGTTGGCTGCTG |
| 3’RACE-1(Outer) | ctctttctattccagggattgcag | Universal primer in SMARTer kit |
| 3’RACE-2(Inner) | CTAATACGACTCACTATAGGGC | Universal primer in SMARTer kit |

**Supplementary Table 7.** **Full-length nucleotide sequence for IL6-AS1(see also Supplementary Fig. 2)**

CATGGGATGCGCACCGGGCCCTTCAACCCCATCGCCAAGCCTACCCACCTCCTTTCTCAGGGCTGAGATGCCGTCGAGGATGTACCGAATTTGTTTGTCAATTCGTTCTGAAGAGCCCCCCAAGCCTGGGATTATGAAGAAGGTAATACTACCAGTCATCTGAGTTCTTCTGTGTTCTGGCTCTCCCTGTGAGCGGCTGTTGTAGAACTGCCTGGCCATCCTCAAATTTCGTGCAGTTACTTCAAGGCGTCTCCAGGTGGAGTGTGTGACTCCTTTTTGCCACCGCGTGGCTTCTGCCACTTTCTTCACCTGCTTCAGCCCACTTAGAGGAGGTTGGCTGCTGCCCTGCCCTCTCCCAAGTGACCCCTTGGGAATCCTCCCAGTGAGGACTTTGCTCTCTGTGCCAGAGGTTTGCCTTGTGCTGCCAGTGCTCTCCCCATTGCCACTGAGTCTCTTCACCACTGTTTCACTGGGGCACCTGCATGGGCCCCAGGTCTCCTGGAGCCCTGAAATTACTGAAGCCCACTTGGTTCAGGGCAGAAAGGGGGAGAATACTACTCACCTCTTTGTTGGGGATGTCAAAGGAGGACCTTGTGGCATCTTGCAGAGTTTGTGTTTATCGCTCCCTCTCCCTGTAAGTCTTGATTTGAAATCAAAAGAAGCTAGAAGGAAACTACGGACGCAGGCACGGCTCTAGGCTCTGAATCTGCTTCCGCGTCGGCACCCAAGAATTTCTTATTGGGTCAAGCTGGTTCAGAAGCCCTCTTCCCTCAGGATGGTGTCTCTTGCAGGAGGATCCTTGGTCTCATGCAAATGGGCTCTAGACTAGGTTGTCCCTCCAGTCTCCAGAGACCCAGACAGCTCTGAGATGGCTTCAGGCTGAAACCAGACCCTTGCACAACACCAAAACACTTATTTACCAAACATGGTGTTACCTTCACAATCGGTTTCTTTGCTTTGCTAAGAAGAGATCTCTTCAAGATCGATAAAACAGTGACCTCTGTTGGGCATTTACTCAAGTTTTGTTTCGTGCATAACATTTCAGGACCCGCCTGTTGGGGAAAGTGAGGTCATCCATTCTTCACCGATTGTCTAAACAGAGACGGTGGTCCTCTGCCGCGGTGGGATTCTTTGGTGTGAAAATGAAAATGGGGGAGGATGGCTGGATGGTTTCATTTTTAGTGCTCTCCTGTCTTAAGCAACGTAGACACTCCTGAACCAAGTTCTCTTTCCTGCATGAAACGAAGCCACTGCTCCCAGCTCCGCAGCCGTGCACTGTGATCCGTCTATGTAATGTGAGCTTGCTTTTACTCAACTATCTATTTTTTAGAAAATACAATTCAATAAATTTCAAGTACTTGAACTGAGTGACACAATACAACACACAGATTTATCTTTTCAATAAAAAGTGTTTTTTAAATAGGAAAAAAAAAAAAAAAAAAAA

**Supplementary Table 8.** **ChIRP probe for IL6-AS1**

| **ChIRP probe** | **primer** |
| --- | --- |
| IL6-AS1 p1 | CATCCTCGACGGCATCTCAG |
| IL6-AS1 p2 | CACACACTCCACCTGGAGAC |
| IL6-AS1 p3 | GGTGAAGAGACTCAGTGGCA |
| IL6-AS1 p4 | TCAAGACTTACAGGGAGAGG |
| IL6-AS1 p5 | GTCTCTGGAGACTGGAGGGA |
| IL6-AS1 p6 | GTCCTGAAATGTTATGCACGA |
